# Consecutive hydrazino-Ugi-azide reactions: synthesis of acylhydrazines bearing 1,5-disubstituted tetrazoles

**DOI:** 10.3762/bjoc.13.256

**Published:** 2017-12-05

**Authors:** Angélica de Fátima S Barreto, Veronica Alves dos Santos, Carlos Kleber Z Andrade

**Affiliations:** 1 Laboratório de Química Metodológica e Orgânica Sintética, Instituto de Química, Universidade de Brasília, 70910-970, Brasília-DF, Brazil

**Keywords:** acylhydrazines, consecutive Ugi reactions, 1,5-disubstituted tetrazoles, isocyanide-based multicomponent reactions (IMCRs), Ugi-azide reaction

## Abstract

Isocyanide-based multicomponent reactions (IMCRs) allow the construction of relatively complex molecules through a one-pot synthesis. The combination of IMCRs in a consecutive or sequential fashion further extends the complexity of the molecules obtained. Herein, we report the efficient application of this approach to the synthesis of acylhydrazines bearing 1,5-disubstituted tetrazoles. Our strategy was accomplished in only three steps: first, a one-pot hydrazino-Ugi-azide four-component reaction; second a hydrazinolysis and finally an additional hydrazino-Ugi-azide reaction. This sequence provides the title compounds in moderate to excellent yields. The products synthesized herein contain functional groups within their structures that can be easily modified to obtain new acylhydrazino 1,5-disubstituted tetrazoles.

## Introduction

Tetrazoles are extensively studied, useful non-natural heterocyclic skeletons with the highest nitrogen content among the stable heterocycles [1–2]. The tetrazole-ring system has a variety of applications in organic chemistry, coordination chemistry, and agriculture and, in particular, it displays a wide range of biological properties such as analgesic, anti-inflammatory, antiviral, anticancer, among others [3–5].

**Figure 1:**
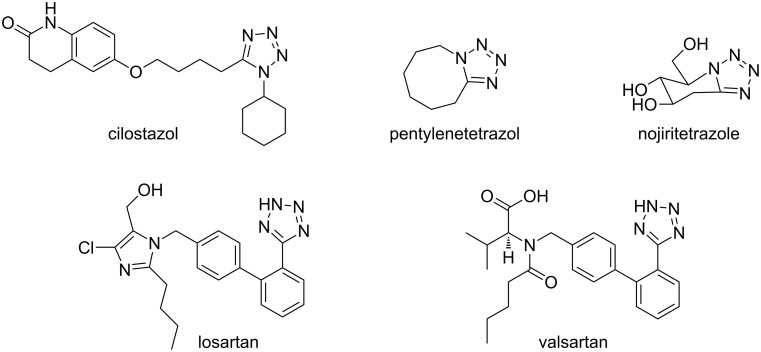
Tetrazole-containing drugs.

The tetrazole nucleus most widely described in the literature is the 1,5-disubstituted tetrazole [6–7] because it presents a wide range of pharmacological activities. For instance, cilostazol (anti-inflammatory), pentylenetetrazol (circulatory and respiratory stimulant) and nojiritetrazole (antidiabetic) are drugs containing the 1,5-disubstituted tetrazole nucleus, along with the pharmaceutically important tetrazoles losartan and valsartan, which are used as angiotensin receptor blockers (Figure 1) [8].

In recent years, the synthesis of 1,5-disubstituted tetrazoles has attracted the attention of several research groups around the world not only for their important pharmacological activities but also for being an excellent mimic of the *cis*-amide bond. Furthermore, they can be used to pre-organize the amide bonds of peptides [9].

Most methods for the synthesis of tetrazoles have limitations that include multistep syntheses of starting materials and/or the use of expensive reagents. The most commonly used synthesis of 1,5-disubstituted tetrazoles are intermolecular cycloaddition reactions and isocyanide-based multicomponent reactions (IMCRs). Indeed, the Ugi-multicomponent reaction using TMSN_3_ (trimethylsilyl azide) as acid component, originally reported by Ugi in 1961 [10], is one of the best and most general methods for the synthesis of 1,5-disubstituted tetrazoles due to the intrinsic generation of molecular diversity [11–15]. As the Ugi reaction also requires an amine component, we envisaged that reactions of hydrazino compounds (RNHNH_2_) with TMSN_3_ could furnish useful compounds bearing both the hydrazino and the tetrazole moieties.

The term ‘hydrazino-Ugi’ was first proposed for the synthesis of hydrazinopeptides [16] in 2010. The application of consecutive hydrazino-Ugi reactions has also been described in the literature [17]. Besides this, the introduction of the hydrazino group into several classes of compounds presents several advantages. For instance, hydrazinopeptides comprise a class of peptidomimetics with promising biological and conformational activities [18–21]. It is worth mentioning that in such compounds the so-called "hydrazino-turns" are formed through intramolecular hydrogen bonding between the hydrazino groups. The resulting unique secondary structures can improve the proteolytic stability of these compounds [22]. An acylhydrazine (hydrazide) was first reported in Ugi reactions back in 1961 [23] and also some natural products contain this moiety, such as the vitamin B6 antagonist linatine [24] and the antibiotic negamycin, active against Gram-negative bacteria [25], among others [26]. Furthermore, trisubstituted acylhydrazines were found to serve as tertiary amide bioisosters [27]. Therefore, it is highly desirable to have a method that allows an easy incorporation of hydrazino groups into more complex molecules. In this respect, Dömling et al. have recently described the synthesis of α-hydrazino amides using *N*-hydroxyimides as the acid component in Ugi reactions [28].

As part of our continuing efforts in using consecutive multicomponent reactions to obtain novel molecules in a reduced number of steps [29–31], herein we describe a concise and efficient strategy for the synthesis of acylhydrazino bistetrazoles through hydrazino-Ugi-azide reactions in only three steps. During the course of our work, Dömling et al. reported the synthesis of hydrazinotetrazoles through a different approach focused on their postcyclization strategy [32].

## Results and Discussion

**Scheme 1:**
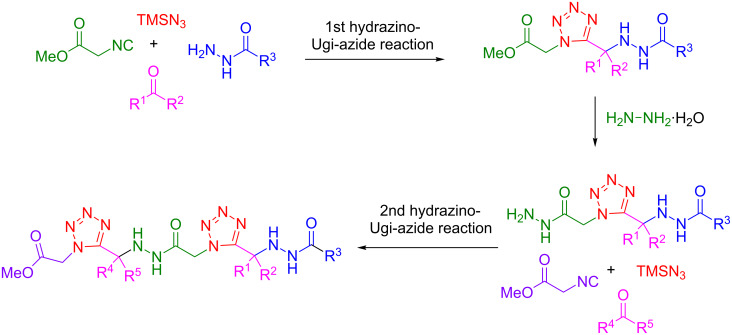
Synthesis of acylhydrazino bis(1,5-disubstituted tetrazoles) through two hydrazino-Ugi-azide reactions and a hydrazinolysis step.

Two multicomponent reactions were used for the synthesis of acylhydrazino bis(1,5-disubstituted tetrazoles) as outlined in Scheme 1. The approach presented here is initially based on a hydrazino-Ugi-azide reaction, followed by a hydrazinolysis step and a second hydrazine-Ugi-azide reaction to provide the desired acylhydrazino bis(1,5-disubstituted tetrazoles). Recently we have reported the synthesis of acylhydrazino-peptomers by a similar strategy [31].

**Scheme 2:**
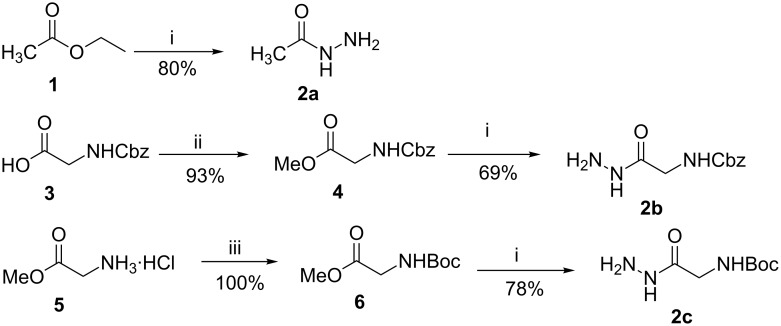
Synthesis of hydrazides **2a**–**c**. Reagents and conditions: (i) N_2_H_4_·H_2_O, EtOH, reflux, 2–3 h; (ii) CH_3_I, NaHCO_3_, DMF, rt, 46 h; (iii) (Boc)_2_O, NaOH, dioxane/H_2_O, overnight [31].

The hydrazides **2a–c** were prepared by the reaction of esters **1**, **4** and **6** with hydrazine monohydrate, following a known procedure (Scheme 2) [31].

**Scheme 3:**
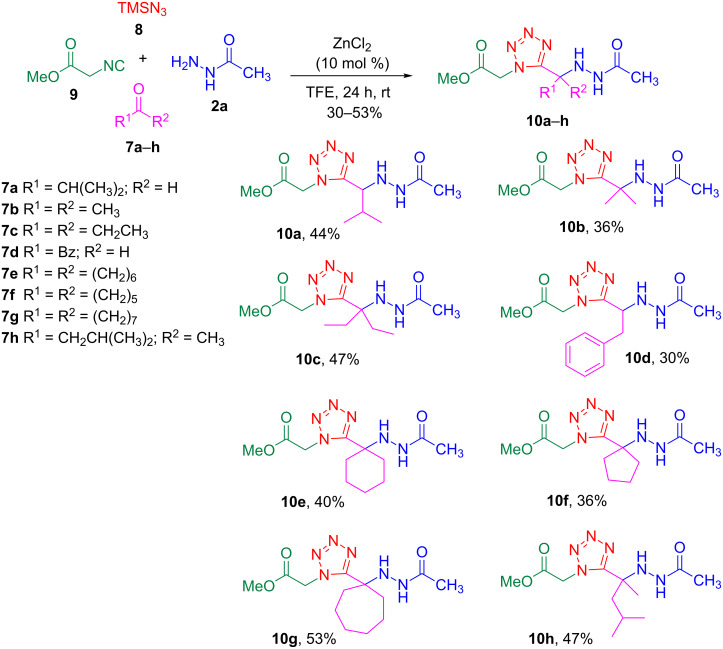
Synthesis of acylhydrazino 1,5-disubstituted tetrazoles **10a**–**h** through multicomponent reactions involving **2a**.

**Scheme 4:**
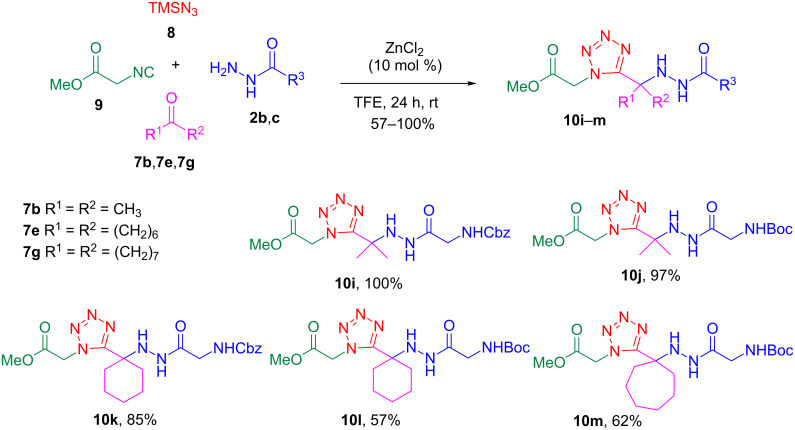
Synthesis of acylhydrazino 1,5-disubstituted tetrazoles **10i**–**m** through multicomponent reactions involving **2b** or **2c**.

To access the acylhydrazino-tetrazoles, the hydrazides **2a**–**c** were subjected to a multicomponent reaction comprising an aldehyde or ketone **7a**–**h**, trimethylsilyl azide (TMSN_3_, **8**), methyl isocyanoacetate (**9**) and ZnCl_2_ (10 mol %) in trifluoroethanol (TFE) at room temperature for 24 h. The presence of catalytic amounts of zinc chloride as Lewis acid in this reaction had already been reported to improve the yields [33]. Indeed, initial studies without the catalyst afforded the desired products **10a** in only 28% yield (120 °C, 30 min, MW, TFE) and **10j** in 68% yield (rt, 24 h, TFE). As solvent, TFE was found to be the best choice for the reactions. The results summarized in Scheme 3 show that various ketones and aldehydes generated the respective products under the optimized conditions when acetyl hydrazide (**2a**) was employed, albeit in moderate yields (30–53%). Nevertheless, the yields were considerably higher for Cbz-glycine hydrazide (**2b**) and Boc-glycine hydrazide (**2c**) when compared to **2a** (Scheme 4).

**Scheme 5:**
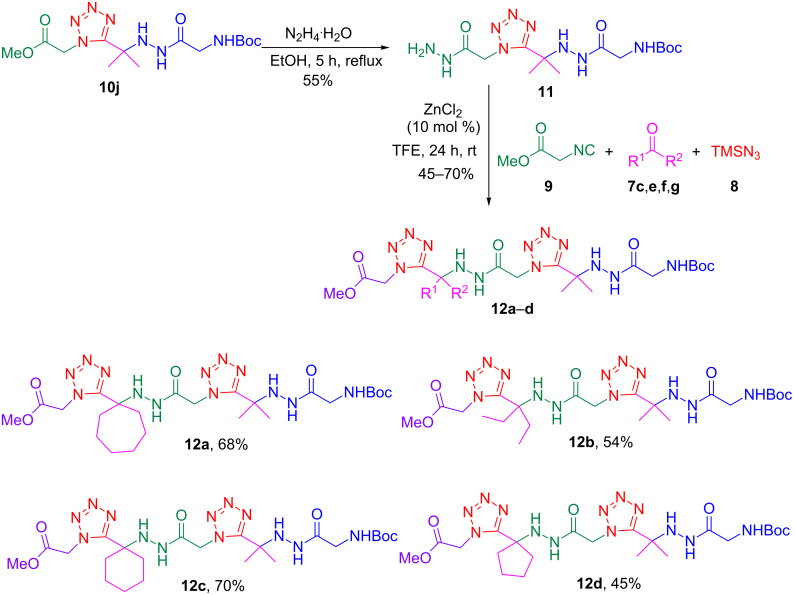
Synthesis of acylhydrazino bis(1,5-disubstituted tetrazoles) **12a**–**d**.

Next, a second hydrazino-Ugi-azide reaction was carried out to obtain the acylhydrazino bistetrazoles. For this purpose, we chose hydrazine tetrazole **10j** to continue the synthesis, as it was obtained in excellent yield, it shows distinct NMR spectra and offers the possibility of further functionalization after removal of the Boc protecting group. Therefore, compound **10j** was subjected to hydrazinolysis with hydrazine monohydrate as already described to give the corresponding hydrazide **11** (Scheme 5). The latter compound was then subjected to the hydrazine-Ugi-azide reaction with different ketones, to yield the corresponding acylhydrazino bis(1,5-disubstituted tetrazoles) **12a**–**d** in moderate to good yields (45–70%). At this stage, only symmetrical ketones were used to facilitate the product characterization by NMR analysis. Indeed the ^1^H and ^13^C NMR spectra were in full agreement with the proposed structures. Of note are the characteristic C=N tetrazole resonances that are observed between 155–158 ppm in the ^13^C NMR spectra. In addition, HRMS data supported the product structures.

**Scheme 6:**
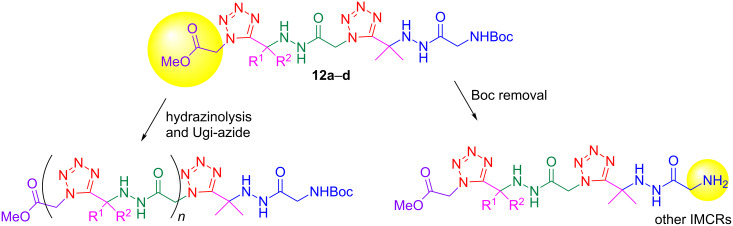
Possible postmodification reactions of the acylhydrazino bis(1,5-disubstituted tetrazoles) **12a**–**d**.

The obtained acylhydrazino bis(1,5-disubstituted tetrazoles) **12a**–**d** can be further functionalized on either or both termini after ester cleavage and/or Boc deprotection. So, further Ugi/Ugi-azide reactions allow elongating the acylhydrazino bis(1,5-disubstituted tetrazoles) main chain (Scheme 6). For instance, the ester terminus can be submitted to another hydrazinolysis to install a new tetrazole nucleus after an Ugi-azide reaction.

## Conclusion

In summary, the approach developed herein allows the synthesis of a wide range of hydrazino bis(1,5-disubstituted tetrazoles) in only three steps. The procedure offers several advantages, such as high atom-economy, a simple synthetic procedure with an easy work-up and ready access to highly functionalized compounds in a low number of steps. In addition, the obtained compounds allow further modification reactions.

## Supporting Information

File 1 Detailed experimental procedures, NMR and mass spectra.
