# Supplementary material for: Consecutive hydrazino-Ugi-azide reactions: synthesis of acylhydrazines bearing 1,5-disubstituted tetrazoles
Source: Beilstein J Org Chem. 2017 Dec 5;13:2596–602. doi: 10.3762/bjoc.13.256 (PMC5727845; doi:10.3762/bjoc.13.256)
Supplement: File 1 — Detailed experimental procedures, NMR and mass spectra. [file Beilstein_J_Org_Chem-13-2596-s001.pdf]

Supporting Information

for

Consecutive hydrazino-Ugi-azide reactions: synthesis of acylhydrazines bearing 1,5-disubstituted tetrazoles

Angélica de Fátima S. Barreto*, Veronica Alves dos Santos, and Carlos Kleber Z. Andrade*

Address: Laboratório de Química Metodológica e Orgânica Sintética, Instituto de Química, Universidade de Brasília, 70910-970, Brasília-DF, Brazil

Email: Angélica de Fátima S. Barreto - angelfsb@yahoo.com.br; Carlos Kleber Z. Andrade - ckleber@unb.br

*Corresponding author

Detailed experimental procedures, NMR and mass spectra

Table of contents

General Information S2
General procedure for the Ugi-tetrazole reaction S2
Spectra of compounds S14–S66

General information

NMR spectra were recorded on a Bruker Ascend instrument using a 5 mm internal diameter probe operating at 600 MHz for ^{1}H and at 150 MHz for ^{13}C in the presence of TMS as internal standard. High resolution ESIMS analyses were carried out on a triple TOF 5600+ (AB Sciex) with internal calibration and direct solution (1 ppm) infusion in positive ion mode. TLC plates were revealed by treatment with a 10% solution of phosphomolybdic acid in ethanol, followed by heating. Melting points were recorded on a Marconi melting point and are uncorrected. Commercially available reagents and solvents were of analytical grade or were purified by standard procedures prior to use. Compounds were analyzed by ^{1}H NMR, ^{13}C NMR and high resolution ESI mass spectra giving data consistent with the proposed structures. Compounds names were given based on ChemDraw® software 7.0.

General procedure for the hydrazino-Ugi-azide reactions: To a stirred solution of hydrazide **2a**–**c** (0.40 mmol) in TFE (1.0 mL) were added successively oxo compound **7a–h** (0.40 mmol, 0.80 mmol when a ketone was used), trimethylsilylazide (**8**, TMS azide, 0.40 mmol), methyl isocyanoacetate (**9**, 0.40 mmol) and $ZnCl_2$ (10 mol %). The resulting mixture was stirred at room temperature for 24 h. The solvent was removed under reduced pressure and the residue was purified by column chromatography to obtain the Ugi tetrazole product.

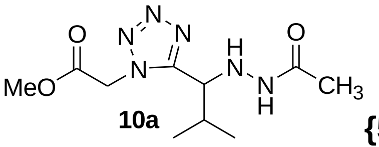

10a

{5-[1-(*N'*-Acetylhydrazino)-2-methylpropyl] tetrazol-1-yl}acetic acid methyl ester (10a): Prepared following the general procedure using acetylhydrazide (**2a**, 0.73 mmol, 0.054 g), isobutyraldehyde (**7a,** 0.73 mmol, 0.053 g), trimethylsilyl azide (**8**, 0.73 mmol, 0.084 g), methyl isocyanoacetate (**9**, 0.73 mmol, 0.066 mL) and $ZnCl_2$ (0.073 mmol, 0.010 g). Purification by column chromatography (CH_2Cl_2 → 2% MeOH/CH_2Cl_2) furnished product **10a** in 44% yield (0.086 g, 0.32 mmol) as a yellow oil. R_f (CH_2Cl_2/MeOH 5%) 0.28.

^{1}H NMR (600 MHz, $CDCl_3$) δ 7.47 (br s, 1H), 5.39 (s, 2H), 5.06 (br s, 1H), 4.31 (d, J = 8.8 Hz, 1H), 3.85 (s, 3H), 2.08-2.01 (m, 1H), 1.88 (s, 3H), 1.21 (d, J = 6.6 Hz, 3H), 0.81 (d, J = 6.6 Hz, 3H). ^{13}C NMR (150 MHz, $CDCl_3$): δ 169.8, 167.6, 154.9, 63.0, 53.4, 48.8, 30.7, 20.9, 19.6, 19.2. HRMS (ESI) *m/z*: calcd. for $[M+Na]^+$ $C_{10}H_{18}N_6O_3Na$: 293.1338; found: 293.1342.

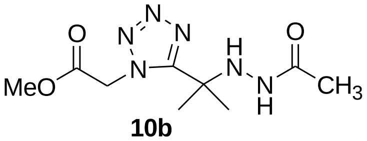

10b

{5-[1-(*N'*-Acetylhydrazino)-1-methylethyl]-tetrazol-1-yl}acetic acid methyl ester (10b): Prepared following the general procedure using acetylhydrazide (**2a**, 0.73 mmol, 0.054 g), acetone (**7b**, 0.146 mmol, 0.107 mL), trimethylsilyl azide (**8**, 0.73 mmol, 0.084 g), methyl isocyanoacetate (**9**, 0.73 mmol, 0.066 mL) and $ZnCl_2$ (0.073 mmol, 0.010 g). Purification by column chromatography (CH_2Cl_2 → 2% MeOH/CH_2Cl_2) furnished product **10b** in 36% yield (0.067 g, 0.26 mmol) as a white solid. Mp 112–114 °C; R_f (CH_2Cl_2/MeOH 5%) 0.34.

^{1}H NMR (600 MHz, $CDCl_3$) δ 7.18 (br s, 1H), 5.47 (s, 2H), 4.98 (br s, 1H), 3.86 (s, 3H), 1.92 (s, 3H), 1.63 (s, 6H). ^{13}C NMR (150 MHz, $CDCl_3$): δ 169.8, 168.2, 158.3, 57.4, 53.4, 49.7, 24.8, 20.8. HRMS (ESI) *m/z*: calcd. for $[M+Na]^+$ $C_9H_{16}N_6O_3Na$: 279.1182; found: 279.1184.

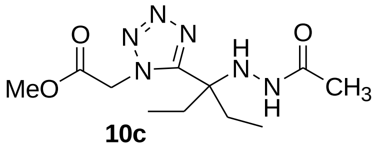

10c

{5-[1-(*N'*-Acetylhydrazino)-1-ethylpropyl]-tetrazol-1-yl}acetic acid methyl ester (10c): Prepared following the general procedure using acetylhydrazide (**2a**, 0.40 mmol, 0.030 g), 3-pentanone (**7c**, 0.40 mmol, 0.034 g), trimethylsilyl azide (**8**, 0.40 mmol, 0.046 g), methyl isocyanoacetate (**9**, 0.40 mmol, 0.036 mL) and $ZnCl_2$ (0.040 mmol, 0.0054 g). Purification by column chromatography (CH_2Cl_2 → 2% MeOH/CH_2Cl_2) furnished product **10c** in 47% yield (0.053 g, 0.19 mmol) as a white solid. Mp 130–132 °C; R_f (CH_2Cl_2/MeOH 5%) 0.31.

^{1}H NMR (600 MHz, $CDCl_3$) δ 7.08 (br s, 1H), 5.54 (s, 2H), 5.26 (br s, 1H), 3.84 (s, 3H), 2.04-1.98 (m, 2H), 1.96-1.90 (m, 2H), 1,89 (s, 3H), 0.88 (t, *J* = 7.0 Hz, 6H). ^{13}C NMR (150 MHz, $CDCl_3$): δ 169.1, 168.4, 157.2, 63.6, 53.4, 49.9, 24.9, 21.0, 7.2. HRMS (ESI) *m/z*: calcd. for $[M+Na]^+$ $C_{11}H_{20}N_6O_3Na$: 307.1495; found: 307.1500.

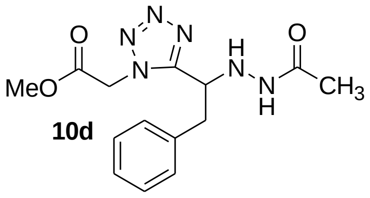

10d

{5-[1-(*N'*-Acetylhydrazino)-2-phenylethyl]-tetrazol-1-yl}acetic acid methyl ester (10d): Prepared following the general procedure using acetylhydrazide (**2a**, 0.73 mmol, 0.054 g), phenylacetaldehyde (**7d**, 0.73 mmol, 0.098 g), trimethylsilyl azide (**8**, 0.73 mmol, 0.084 g), methyl isocyanoacetate (**9**, 0.73 mmol, 0.066 mL) and $ZnCl_2$ (0.073 mmol, 0.010 g). Purification by column chromatography (CH_2Cl_2 → 2% MeOH/CH_2Cl_2) furnished product **10d** in 30% yield (0.069 g, 0.22 mmol) as a yellow oil. R_f (CH_2Cl_2/MeOH 5%) 0.39.

^{1}H NMR (600 MHz, $CDCl_3$) δ 7.50 (s, 1H), 7.30-7.27 (m, 3H), 7.08-7.06 (m, 2H), 4.94 and 4.91 (2s, 1H, rotamers), 4.87 (t, *J* = 7.0 Hz, 1H), 4.79 and 4.76 (2s, 1H, rotamers), 3.79 (s, 3H), 3.23-3.15 (m, 2H), 1.85 (s, 3H). ^{13}C NMR (150

MHz, $CDCl_3$): δ 170.1, 167.2, 155.1, 135.0, 129.2, 128.9, 127.6, 57.1, 53.3, 48.0, 38.9, 20.9. HRMS (ESI) *m/z*: calcd. for $[M+Na]^+$ $C_{14}H_{18}N_6O_3Na$: 341.1338; found: 341.1344.

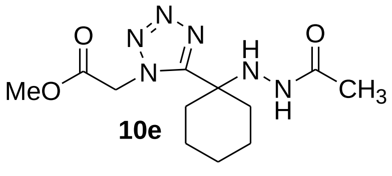

10e

{5-[1-(*N'*-Acetylhydrazino)cyclohexyl]-tetrazol-1-yl}acetic acid methyl ester (10e): Prepared following the general procedure using acetylhydrazide (**2a**, 0.73 mmol, 0.054 g), cyclohexanone (**7e**, 0.73 mmol, 0.072 g), trimethylsilyl azide (**8**, 0.73 mmol, 0.084 g), methyl isocyanoacetate (**9**, 0.73 mmol, 0.066 mL) and $ZnCl_2$ (0.073 mmol, 0.010 g). Purification by column chromatography (CH_2Cl_2 → 2% $MeOH/CH_2Cl_2$) furnished product **10e** in 40% yield (0.093 g, 0.29 mmol) as a white solid. Mp 116–118 °C, R_f (CH_2Cl_2/MeOH 5%) 0.32.

^{1}H NMR (600 MHz, $CDCl_3$) δ 7.17 (d, *J* = 5.9 Hz, 1H), 5.51 (s, 2H), 5.30 (d, *J* = 7.3 Hz, 1H), 3.84 (s, 3H), 2.19-2.15 (m, 2H), 1.89 (s, 3H), 1.86-1.82 (m, 2H), 1.77-1.71 (m, 2H), 1.65-1.59 (m, 2H), 1.51-1.48 (m, 2H). ^{13}C NMR (150 MHz, $CDCl_3$): δ 169.5, 168.1, 157.6, 59.5, 53.4, 49.8, 33.1, 24.9, 21.8, 20.9. HRMS (ESI) *m/z*: calcd. for $[M+Na]^+$ $C_{12}H_{20}N_6O_3Na$: 319.1495; found: 319.1501.

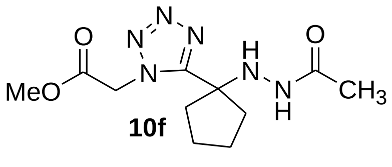

10f

{5-[1-(*N'*-Acetylhydrazino)cyclopentyl]-tetrazol-1-yl}acetic acid methyl ester (10f): Prepared following the general procedure using acetylhydrazide (**2a**, 0.73 mmol, 0.054 g), cyclopentanone (**7f**, 0.73 mmol, 0,061 g), trimethylsilyl azide (**8**, 0.73 mmol, 0.084 g), methyl isocyanoacetate (**9**, 0.73 mmol, 0.066 mL) and $ZnCl_2$ (0.073 mmol, 0.010 g). Purification by column chromatography (CH_2Cl_2 → 2% $MeOH/CH_2Cl_2$) furnished product **10f** in 36% yield (0.074 g, 0.26 mmol) as a yellow solid. Mp 105–107 °C; R_f (CH_2Cl_2/MeOH 5%) 0.36.

^1H NMR (600 MHz, $CDCl_3$) δ 7.21 (d, *J* = 7.5 Hz, 1H), 5.41 (s, 2H), 5.15 (d, *J* = 7.7 Hz, 1H), 3.84 (s, 3H), 2.29-2.24 (m, 2H), 2.08-2.04 (m, 1H), 1.97-1.92 (m, 2H), 1.90 (s, 3H), 1.84-1.77 (m, 2H). ^{13}C NMR (150 MHz, $CDCl_3$): δ 170.0, 168.1, 158.4, 67.6, 53.4, 49.3, 35.0, 27.0, 24.8, 23.9, 20.9. HRMS (ESI) *m/z*: calcd. for $[M+Na]^+$ $C_{11}H_{18}N_6O_3Na$: 305.1338; found: 305.1350.

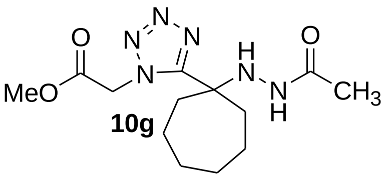

10g

{5-[1-(*N'*-Acetylhydrazino)cycloheptyl]-tetrazol-1-yl}acetic acid methyl ester (10g): Prepared following the general procedure using acetylhydrazide (**2a**; 0.40 mmol, 0.030 g), cycloheptanone (**7g**, 0.40 mmol, 0.045 g), trimethylsilyl azide (**8**, 0.40 mmol, 0.046 g), methyl isocyanoacetate (**9**, 0.40 mmol, 0.036 mL) and $ZnCl_2$ (0.040 mmol, 0.005 g). Purification by column chromatography (CH_2Cl_2 → 2% $MeOH/CH_2Cl_2$) furnished product **10g** in 53% yield (0,066 g, 0.21 mmol) as a white solid. Mp 102–104 °C; R_f (CH_2Cl_2/MeOH 5%) 0.44.

^1H NMR (600 MHz, $CDCl_3$) δ 7.17 (br s, 1H), 5.49 (s, 2H), 5.24 (d, *J* = 8.1 Hz, 1H), 3.85 (s, 3H), 2.32-2.26 (m, 2H), 1.97-1.93 (m, 2H), 1.90 (s, 3H), 1.70-1.66 (m, 4H), 1.61-1.56 (m, 4H). ^{13}C NMR (150 MHz, $CDCl_3$): δ 169.6, 168.6, 158.8, 63.8, 53.5, 49.9, 35.2, 30.4, 22.3, 20.9. HRMS (ESI) *m/z*: calcd. for $[M+Na]^+$ $C_{13}H_{22}N_6O_3Na$: 333.1651; found: 333.1650.

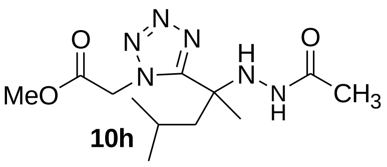

10h

{5-[1-(*N'*-Acetyl-hydrazino)-1,3-dimethyl-butyl]-tetrazol-1-yl}-acetic acid methyl ester (10h): Prepared following the general procedure using acetylhydrazide (**2a**, 0.40 mmol, 0.030 g), methyl isobutyl ketone (**7h**, 0.40 mmol, 0.040 g), trimethylsilyl azide (**8**, 0.40 mmol, 0.046 g),

methyl isocyanoacetate (**9**, 0.40 mmol, 0.036 mL) and $ZnCl_2$ (0.040 mmol, 0.005 g). Purification by column chromatography (CH_2Cl_2 → 2% MeOH/CH_2Cl_2) furnished product **10h** in 47% yield (0.056 g, 019 mmol) as a white solid. Mp 94–96 °C; R_f (CH_2Cl_2/MeOH 5%) 0.42.

^{1}H NMR (600 MHz, $CDCl_3$) δ 7.15 (br s, 1H), 5.54 (d, *J* = 17.0 Hz, 1H), 5.38 (s, *J* = 17.0 Hz, 1H), 5.03 (br s, 1H), 3.86 (s, 3H), 1.91 (s, 3H), 1.72 (s, 3H), 1.70-1.66 (m, 3H), 0.97 (d, *J* = 6.2 Hz, 3H), 0.52 (d, *J* = 6.2 Hz, 3H). ^{13}C NMR (150 MHz, $CDCl_3$): δ 169.9, 168.4, 157.7, 60.7, 53.5, 49.7, 47.2, 24.7, 24.2, 23.2, 21.2, 20.9. HRMS (ESI) *m/z*: calcd. for $[M+Na]^+$ $C_{12}H_{22}N_6O_3Na$: 321.1651; found: 321.1650.

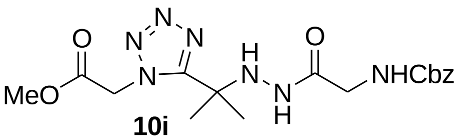

10i

(5-{1-[*N'*-(2-(Benzyloxycarbonyl-amino)acetyl)hydrazino]-1-methylethyl}-tetrazol-1-yl)acetic acid methyl ester (10i): Prepared following the general procedure using Cbz-glycine hydrazide (**2b**, 0.40 mmol, 0.089 g), acetone (**7b**, 0.80 mmol, 0.046 g), trimethylsilyl azide (**8**, 0.40 mmol, 0.046 g), methyl isocyanoacetate (**9**, 0.40 mmol, 0.036 mL) and $ZnCl_2$ (0.040 mmol, 0.005 g). Purification by column chromatography (CH_2Cl_2 → 2% MeOH/CH_2Cl_2) furnished product **10i** in quantitative yield (0.162 g, 0.40 mmol) as a white solid. Mp 120–122 °C; R_f (CH_2Cl_2/MeOH 5%) 0.36.

^{1}H NMR (600 MHz, $CDCl_3$) δ 7.77 (d, *J* = 7.4 Hz, 1H), 7.35-7.29 (m, 5H), 5.47 (s, 2H), 5.45 (br s, 1H), 5.09 (s, 2H), 4.92 (d, *J* = 7.4 Hz, 1H), 3.83 (d, *J* = 5.9 Hz, 2H), 3.80 (s, 3H), 1.60 (s, 6H). ^{13}C NMR (150 MHz, $CDCl_3$): δ 169.1, 168.3, 158.1, 156.5, 136.0, 128.5, 128.2, 128.1, 67.2, 57.5, 53.6, 49.8, 43.3, 24.9. HRMS (ESI) *m/z*: calcd. for $[M+Na]^+$ $C_{17}H_{23}N_7O_5Na$: 428.1658; found: 428.1666.

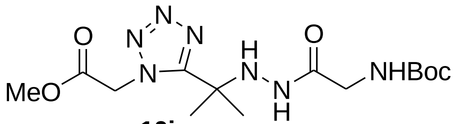

10j

(5-{1-[*N'*-(2-(*tert*-Butoxycarbonylamino)acetyl)hydrazino]-1-methylethyl}-tetrazol-1-yl)acetic acid methyl ester (10j): Prepared following the general procedure using *Boc*-glycine hydrazide (**2c**, 0.40 mmol, 0.076 g), acetone (**7b**, 0.80 mmol, 0.046 g), trimethylsilyl azide (**8**, 0.40 mmol, 0.046 g), methyl isocyanoacetate (**9**, 0.40 mmol, 0.036 mL) and $ZnCl_2$ (0.040 mmol, 0.005 g). Purification by column chromatography (CH_2Cl_2 → 2% MeOH/CH_2Cl_2) furnished product **10j** in 97% yield (0.144 g, 0.39 mmol) as a colorless oil. R_f (CH_2Cl_2/MeOH 5%) 0.34.

^{1}H NMR (600 MHz, $CDCl_3$) δ 7.72 (br s, 1H), 5.47 (s, 2H), 5.04 (br s, 1H), 4.93 (br s, 1H), 3.84 (s, 3H), 3.77 (d, J = 5.9 Hz, 2H), 1.62 (s, 6H), 1.41 (s, 9H). ^{13}C NMR (150 MHz, $CDCl_3$): δ 169.6, 167.9, 158.1, 155.9, 80.2, 57.3, 53.4, 49.8, 42.9, 28.1, 24.8. HRMS (ESI) *m/z*: calcd. for $[M+Na]^+$ $C_{14}H_{25}N_7O_5Na$: 394.1815; found: 394.1814.

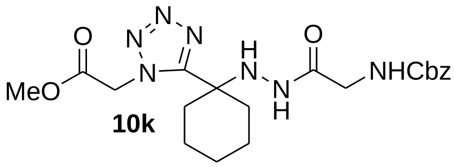

10k

(5-{1-[*N'*-(2-(Benzyloxycarbonylamino)acetyl)hydrazino]-cyclohexyl}-tetrazol-1-yl)acetic acid methyl ester (10k): Prepared following the general procedure using Cbz-glycine hydrazide (**2b**, 0.40 mmol, 0.089 g), cyclohexanone (**7e**, 0.40 mmol, 0.039 g), trimethylsilyl azide (**8**, 0.40 mmol, 0.046 g), methyl isocyanoacetate (**9**, 0.40 mmol, 0.036 mL) and $ZnCl_2$ (0.040 mmol, 0.005 g). Purification by column chromatography (CH_2Cl_2 → 2% MeOH/CH_2Cl_2) furnished product **10k** in 85% yield (0.151 g, 0.34 mmol) as a white solid. Mp 62–64 °C; R_f (CH_2Cl_2/MeOH 5%) 0.42.

^{1}H NMR (600 MHz, $CDCl_3$) δ 7.62 (br s, 1H), 7.36-7.29 (m, 5H), 5.49 (s, 2H), 5.32 (br s, 1H), 5.23 (br s, 1H), 5.08 (s, 2H), 3.80 (s, 5H), 2.18-2.14 (m, 2H), 1.86-1.79 (m, 2H), 1.75-1.69 (m, 2H), 1.64-1.57 (m, 2H), 1.51-1.47 (m, 2H).

^{13}C NMR (150 MHz, $CDCl_3$): δ 168.8, 168.3, 157.5, 156.4, 136.0, 128.5, 128.2, 128.1, 67.2, 59.7, 53.6, 49.9, 43.3, 33.2, 25.0, 21.8. HRMS (ESI) *m/z*: calcd. for $[M+Na]^+$ $C_{20}H_{27}N_7O_5Na$: 468.1971; found: 468.1974.

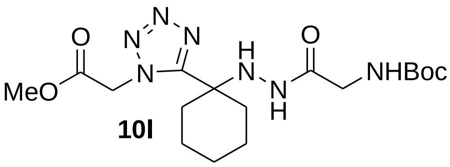

10l

(5-{1-[*N'*-(2-(*tert*-Butoxycarbonylamino)acetyl)hydrazino]-cyclohexyl}-tetrazol-1-yl)acetic acid methyl ester (10l): Prepared following the general procedure using Boc-glycine hydrazide (**2c**, 0.40 mmol, 0.076 g), cyclohexanone (**7e**, 0.40 mmol, 0.039 g), trimethylsilyl azide (**8**, 0.40 mmol, 0.046 g), methyl isocyanoacetate (**9**, 0.40 mmol, 0.036 mL) and $ZnCl_2$ (0.040 mmol, 0.005 g). Purification by column chromatography (CH_2Cl_2 → 3% MeOH/CH_2Cl_2) furnished product **10l** in 57% yield (0.094 g, 0.23 mmol) as a white solid. Mp 68–70 °C; R_f (CH_2Cl_2/MeOH 5%) 0.41.

^1H NMR (600 MHz, $CDCl_3$) δ 7.67 (s, 1H), 5.50 (s, 2H), 5.01 (s, 1H), 3.82 (s, 3H), 3.74 (s, 2H), 2.21-2.17 (m, 2H), 1.87-1.82 (m, 2H), 1.76-1.70 (m, 2H), 1.65-1.59 (m, 2H), 1.51-1.47 (m, 2H), 1.41 (s, 9H). ^{13}C NMR (150 MHz, $CDCl_3$): δ 169.3, 168.1, 157.3, 155.8, 80.4, 59.7, 53.5, 49.9, 43.0, 33.2, 28.2, 24.9, 21.8. HRMS (ESI) *m/z*: calcd. for $[M+Na]^+$ $C_{17}H_{29}N_7O_5Na$: 434.2128; found: 434.2143.

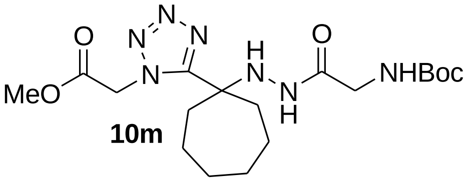

10m

(5-{1-[*N'*-(2-(*tert*-Butoxycarbonylamino)acetyl)hydrazino]-cycloheptyl}-tetrazol-1-yl)acetic acid methyl ester (10m): Prepared following the general procedure using *Boc-*

glycine hydrazide (**2c**, 0.40 mmol, 0.076 g), cycloheptanone (**7g**, 0.40 mmol, 0.045 g), trimethylsilyl azide (**8**, 0.40 mmol, 0.046 g), methyl isocyanoacetate (**9**, 0.40 mmol, 0.036 mL) and $ZnCl_2$ (0.040 mmol, 0.005 g). Purification by column chromatography (CH_2Cl_2 → 4% MeOH/CH_2Cl_2) furnished product **10n** in 62% yield (0.106 g, 0.25 mmol) colorless oil. R_f (CH_2Cl_2/MeOH 5%) 0.50.

^{1}H NMR (600 MHz, $CDCl_3$) δ 7.68 (s, 1H), 5.49 (s, 2H), 5.04 (s, 1H), 3.83 (s, 3H), 3.76 (d, J = 5.5 Hz, 2H), 2.32-2.28 (m, 2H), 1.96-1.92 (m, 2H), 1.68-1.53 (m, 8H), 1.40 (s, 9H). ^{13}C NMR (150 MHz, $CDCl_3$): δ 169.3, 168.2, 158.6, 155.8, 80.3, 63.8, 53.5, 50.8, 49.9, 43.8, 35.3, 30.4, 28.2, 24.3, 22.3. HRMS (ESI) *m/z*: calcd. for $[M+Na]^+$ $C_{18}H_{31}N_7O_5Na$: 448.2284; found: 448.2296.

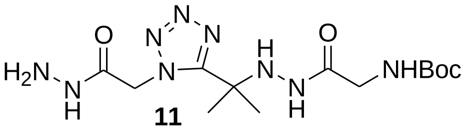

11

{*N'*-[1-(1-Hydrazinocarbonylmethyl-1*H*-tetrazol-5-yl)-1-methylethyl]-hydrazinocarbonylmethyl}carbamic acid *tert*-butyl ester (11): To a solution of compound **10j** (0.371 g, 1.00 mmol) in 2.0 mL of ethanol was added hydrazine hydrate (0.200 g, 4.00 mmol). After refluxing for 5 h, the residue was concentrated in vacuum and purified by column chromatography (CH_2Cl_2 → 10% MeOH/CH_2Cl_2) furnishing product **6** in 55% yield (0.204 g, 0.55 mmol) as a white solid. Mp 44–46 °C; R_f (CH_2Cl_2/MeOH 15%) 0.39.

^{1}H NMR (600 MHz, DMSO-d_6) δ 9.59 (s, 1H), 9.00 (d, J = 5.9 Hz, 1H), 6.95 (t, J = 6.1 Hz, 1H), 5.51 (s, 2H), 5.37 (d, J = 5.9 Hz, 1H), 4.39 (br, 1H), 3.43 (d, J = 5.9 Hz, 2H), 1.46 (s, 6H), 1.35 (s, 9H). ^{13}C NMR (150 MHz, DMSO-d_6): δ 169.1, 165.2, 158.3, 155.8, 78.1, 56.5, 49.5, 34.7, 28.2, 25.1, 18.6, 13.9. HRMS (ESI) *m/z*: calcd. for $[M+Na]^+$ $C_{13}H_{25}N_9O_4Na$: 394.1927; found: 394.1923.

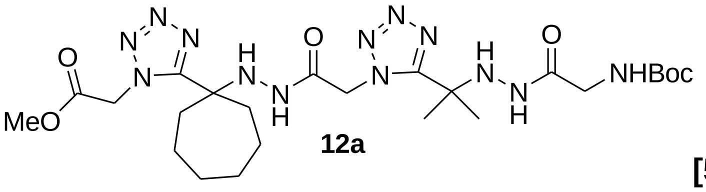

12a

[5-(1-{*N'*-[2-(5-{1-[*N'*-(2-(*tert*-Butoxycarbonylamino)acetyl)hydrazino]-1-methylethyl}-tetrazol-1-yl)acetyl]hydrazino}-cycloheptyl)-tetrazol-1-yl]acetic acid methyl ester (12a): Prepared following the general procedure using hydrazide **11** (0.13 mmol, 0.048 g), cycloheptanone (**7g**, 0.13 mmol, 0.014 g), trimethylsilyl azide (**8**, 0.13 mmol, 0.015 g), methyl isocyanoacetate (**9**, 0.13 mmol, 0.013 mL) and $ZnCl_2$ (0.013 mmol, 0.002 g). Purification by column chromatography (CH_2Cl_2 → 4% MeOH/CH_2Cl_2) furnished product **12a** in 68% yield (0.055 g, 0.090 mmol) as a white solid. Mp 105–107 °C; R_f (CH_2Cl_2/MeOH 10%) 0.52.

^{1}H NMR (600 MHz, DMSO-*d*$_6$) δ 9.59 (d, *J* = 4.4 Hz, 1H), 8.94 (d, *J* = 5.1 Hz, 1H), 6.88 (s, 1H), 5.87 (s, 2H), 5.64 (d, *J* = 4.4 Hz, 1H), 5.45 (s, 2H), 5.27 (d, *J* = 5.5 Hz, 1H), 3.71 (s, 3H), 3.39 (d, *J* = 5.9 Hz, 2H), 2.17-2.13 (m, 2H), 1.96-1.92 (m, 2H), 1.65-1.59 (m, 2H), 1.53-1.48 (m, 4H), 1.41-1.30 (m, 17H). ^{13}C NMR (150 MHz, DMSO-*d*$_6$): δ 169.0, 167.6, 165.6, 158.0 (2C), 155.7, 78.0, 62.4, 56.1, 54.9, 52.7, 49.6, 49.3, 45.7, 41.7, 35.0, 29.6, 28.1, 24.9, 21.9. HRMS (ESI) *m/z*: calcd. for $[M+Na]^+$ $C_{24}H_{41}N_{13}O_6Na$: 630.3200; found: 630.3230.

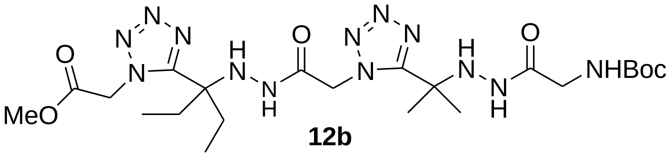

12b

[5-(1-{*N'*-[2-(5-{1-[*N'*-(2-(*tert*-Butoxycarbonylamino)acetyl)hydrazino]-1-methylethyl}-tetrazol-1-yl)acetyl]hydrazino}-1-ethylpropyl)-tetrazol-1-yl]acetic acid methyl ester (12b): Prepared following the general procedure using hydrazide **11** (0.35 mmol, 0.130 g), 3-pentanone (**7c**, 0.35 mmol, 0.030 g), trimethylsilyl azide (**8**, 0.35 mmol, 0.040 g), methyl isocyanoacetate (**9**, 0.35 mmol, 0.032 mL) and

$ZnCl_2$ (0.035 mmol, 0.005 g). Purification by column chromatography (CH_2Cl_2 → 5% MeOH/CH_2Cl_2) furnished product **12b** in 54% yield (0.110 g, 0.19 mmol) as a yellow solid. Mp 101–103 °C; R_f (CH_2Cl_2/MeOH 10%) 0.43.

^{1}H NMR (600 MHz, DMSO-d_6) δ 9.47 (br s, 1H), 8.95 (d, *J* = 5.5 Hz, 1H), 6.88 (t, *J* = 6.1 Hz, 1H), 5.93 (s, 2H), 5.66 (br s, 1H), 5.46 (s, 2H), 5.25 (d, *J* = 5.5 Hz, 1H), 3.70 (s, 3H), 3.39 (d, *J* = 5.9 Hz, 2H), 1.96-1.90 (m, 2H), 1.80-1.74 (m, 2H), 1.35 and 1.33 (2s, 15H), 0.69 (t, *J* = 7.5 Hz, 6H). ^{13}C NMR (150 MHz, DMSO-d_6): δ 169.0, 167.6, 165.7, 158.0, 156.4, 155.7, 78.0, 62.5, 56.0, 52.7, 49.8, 49.3, 45.7, 41.7, 28.1, 24.8, 24.7, 20.0, 7.03. HRMS (ESI) *m/z*: calcd. for $[M+Na]^+$ $C_{22}H_{39}N_{13}O_6Na$: 604.3044; found: 604.3035.

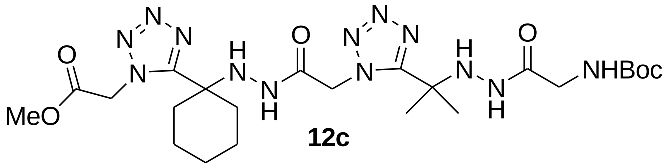

12c

[5-(1-{*N'*-[2-(5-{1-[*N'*-(2-(*tert*-Butoxycarbonylamino)acetyl)hydrazino]-1-methylethyl}-tetrazol-1-yl)acetyl]hydrazino}-cyclohexyl)-tetrazol-1-yl]acetic acid methyl ester (12c): Prepared following the general procedure using hydrazide **11** (0.26 mmol, 0.095 g), cyclohexanone (**7e**, 0.26 mmol, 0.025 g), trimethylsilyl azide (**8**, 0.26 mmol, 0.030 g), methyl isocyanoacetate (**9**, 0.26 mmol, 0.024 mL) and $ZnCl_2$ (0.026 mmol, 0.004 g). Purification by column chromatography (CH_2Cl_2 → 4% MeOH/CH_2Cl_2) furnished product **12c** in 70% yield (0.108 g, 0.18 mmol) as a yellow solid. Mp 119–121 °C; R_f (CH_2Cl_2/MeOH 10%) 0.47.

^{1}H NMR (600 MHz, DMSO-d_6) δ 9.59 (br s,1H), 8.92 (d, *J* = 5.3 Hz, 1H), 6.88 (s, 1H), 5.84 (s, 2H), 5.67 (s, 1H), 5.41 (s, 2H), 5.25 (d, *J* = 5.3 Hz, 1H), 3.70 (s, 3H), 3.40 (d, *J* = 5.9 Hz, 2H), 2.05-1.99 (m, 2H), 1.80-1.77 (m, 2H), 1.69-1.60 (m, 2H), 1.47-1.39 (m, 4H), 1.35 and 1.33 (2s, 15H). ^{13}C NMR (150 MHz, DMSO-d_6): δ 169.0, 167.6, 165.6, 158.0, 156.8, 155.7, 78.0, 58.2, 56.1, 52.7, 49.5, 49.2, 45.7, 41.7, 32.8, 28.1, 24.9, 21.4. HRMS (ESI) *m/z*: calcd. for $[M+Na]^+$ $C_{23}H_{39}N_{13}O_6Na$: 616.3044; found: 616.3033.

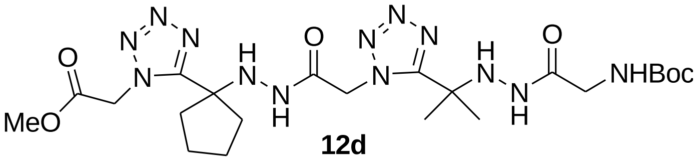

12d

[5-(1-{*N'*-[2-(5-{1-[*N'*-(2-(*tert*-Butoxycarbonylamino)acetyl)hydrazino]-1-methylethyl}-tetrazol-1-yl)acetyl]hydrazino}-cyclopentyl)-tetrazol-1-yl]acetic acid methyl ester (12d): Prepared following the general procedure using hydrazide **11** (0.30 mmol, 0.110 g), cyclopentanone (**7f**, 0.30 mmol, 0.025 g), trimethylsilyl azide (**8**, 0.30 mmol, 0.034 g), methyl isocyanoacetate (**9**, 0.30 mmol, 0.027 mL) and $ZnCl_2$ (0.030 mmol, 0.004 g). Purification by column chromatography (CH_2Cl_2 → 4% MeOH/CH_2Cl_2) furnished product **12d** in 45% yield (0.079 g, 0.14 mmol) as a yellow solid. Mp 114–116 °C; R_f (CH_2Cl_2/MeOH 10%) 0.47.

^{1}H NMR (600 MHz, $CDCl_3$) δ 8.44 (d, *J* = 7.0 Hz, 1H), 8.39 (br s, 1H), 5.47 (s, 2H), 5.41 (s, 2H), 5.27 (br s, 1H), 5.14 (br s, 1H), 4.75 (br s, 1H), 3.86 (s, 3H), 3.77 (d, *J* = 5.9 Hz, 1H), 2.29-2.24 (m, 1H), 2.09-2.02 (m, 1H), 1.95-1.88 (m, 2H), 1.78-1.76 (m, 2H), 1.59 (s, 6H), 1.43 (s, 9H). ^{13}C NMR (150 MHz, $CDCl_3$): δ 169.9, 168.1, 165.6, 158.2, 157.9, 156.0, 80.3, 67.6, 57.5, 53.7, 49.9, 49.4, 42.9, 35.2, 29.7, 28.3, 25.2, 23.8, 14.1. HRMS (ESI) *m/z*: calcd. for $[M+Na]^+$ $C_{22}H_{37}N_{13}O_6Na$: 602.2887; found: 602.2879.

Spectra of compounds

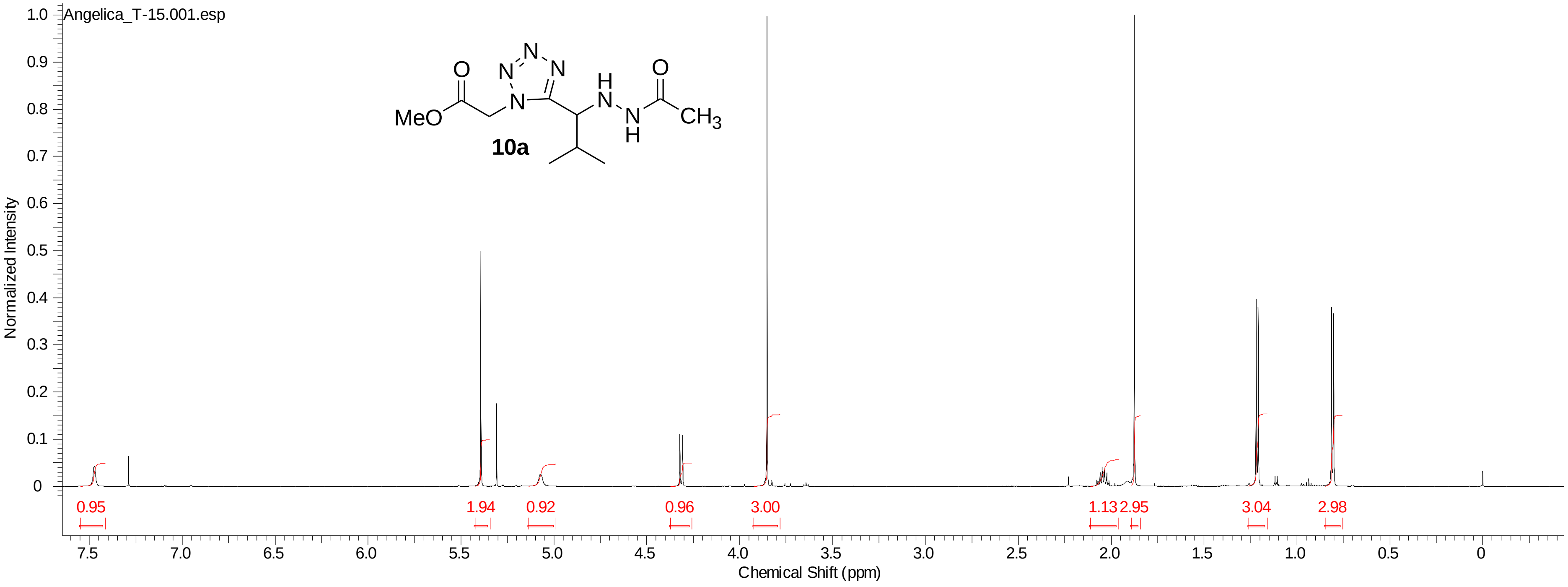

Figure S1: ^{1}H NMR (600 MHz, $CDCl_3$) spectrum of compound **10a**.

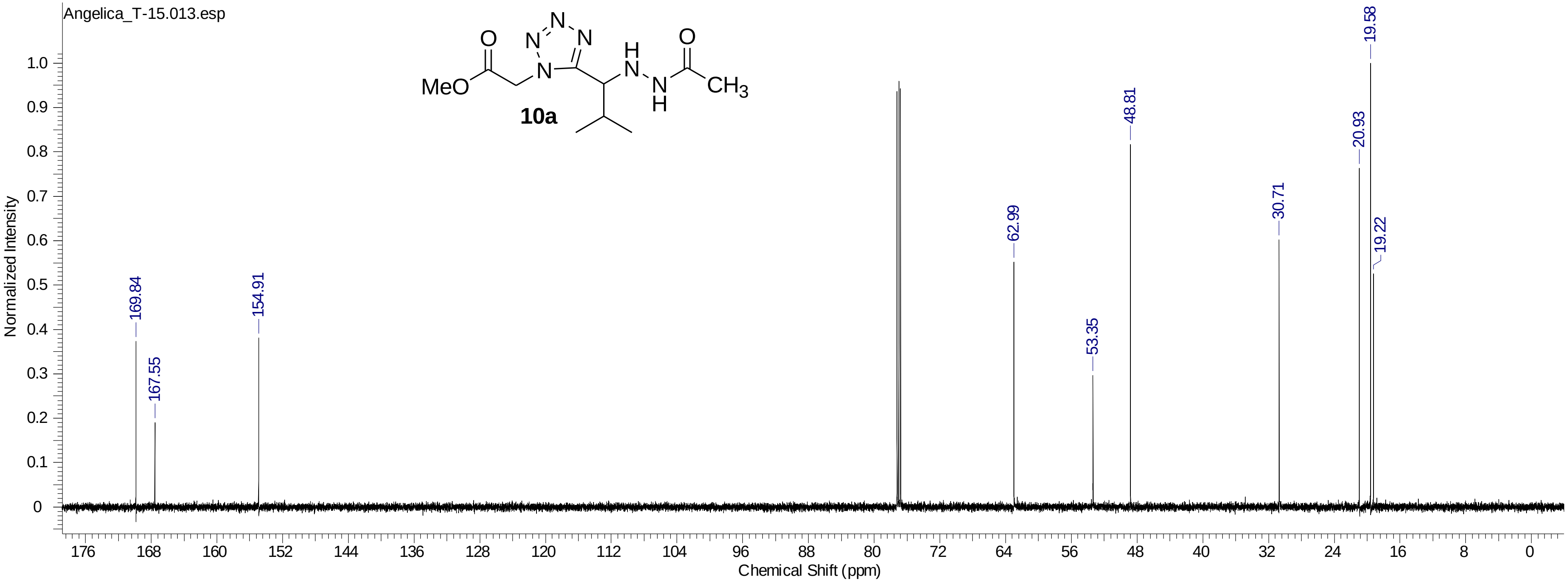

Figure S2: ^{13}C NMR (150 MHz, $CDCl_3$) spectrum of compound **10a**.

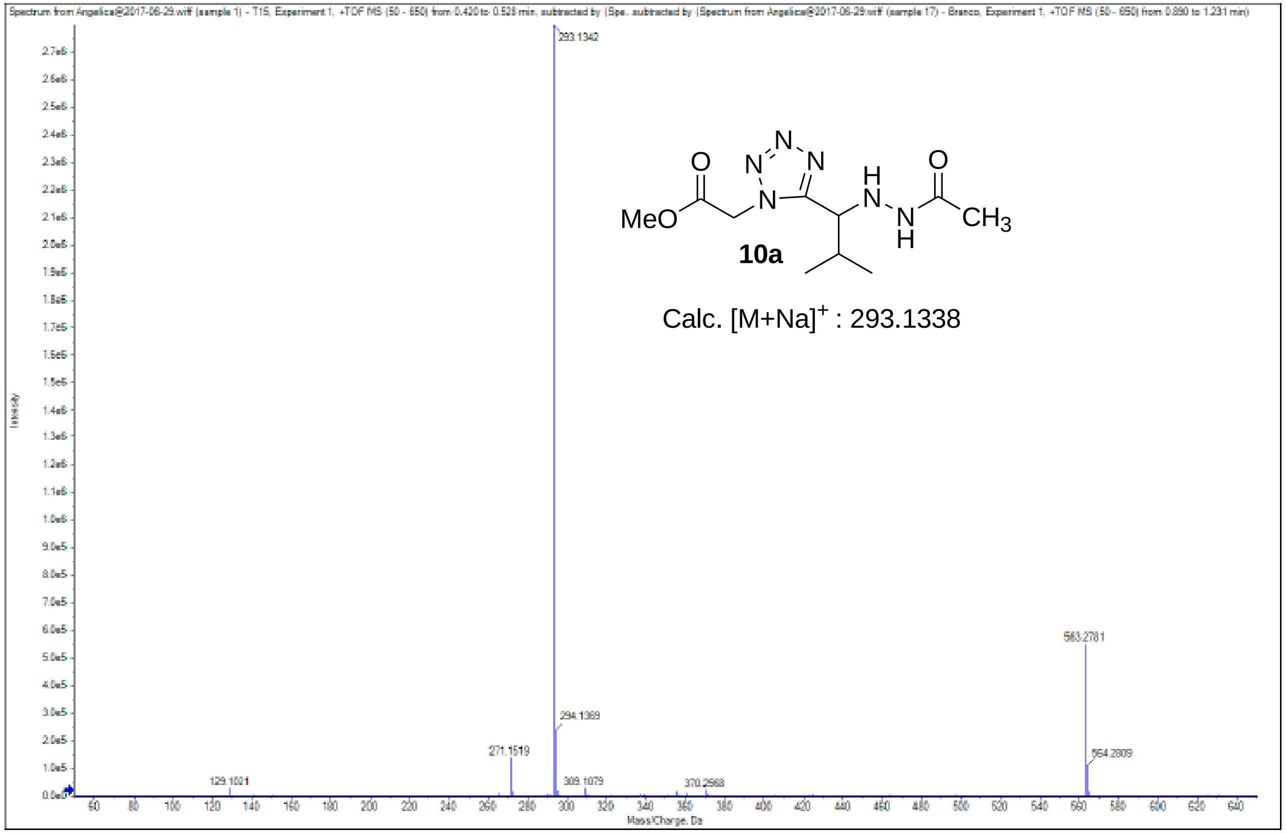

Figure S3: ESI-HRMS of compound **10a.**

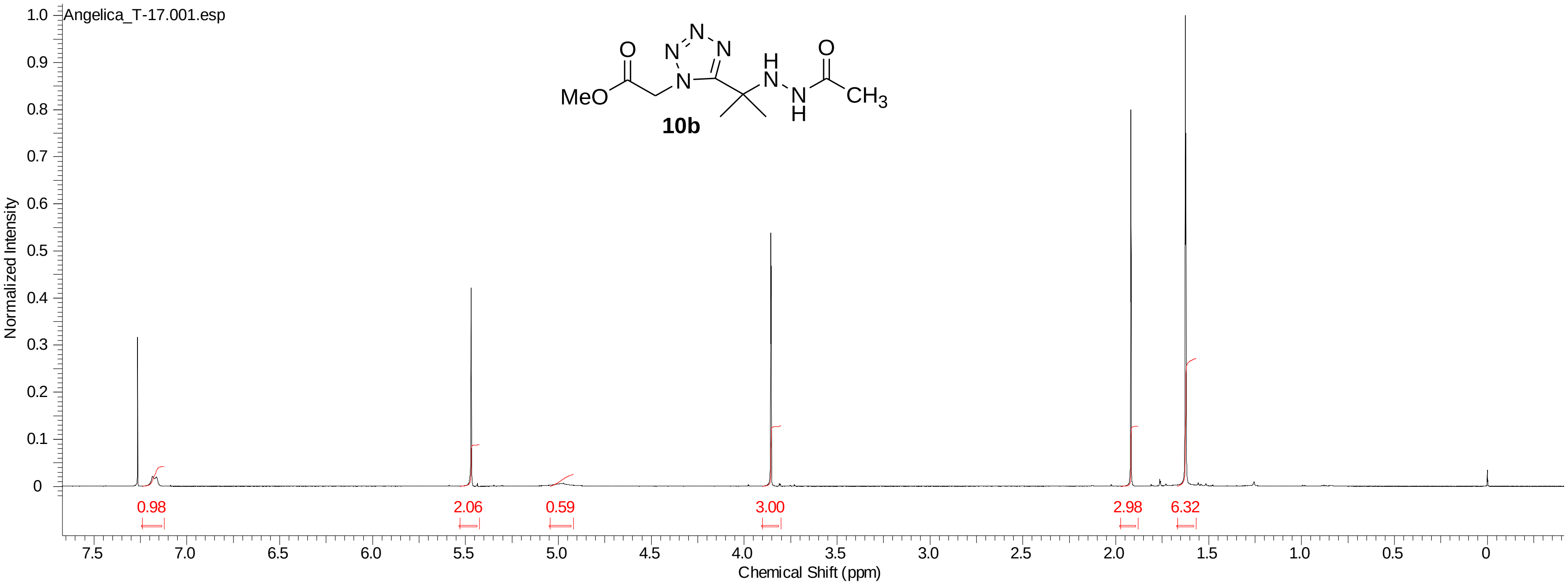

Figure S4: ^{1}H NMR (600 MHz, $CDCl_3$) spectrum of compound **10b**.

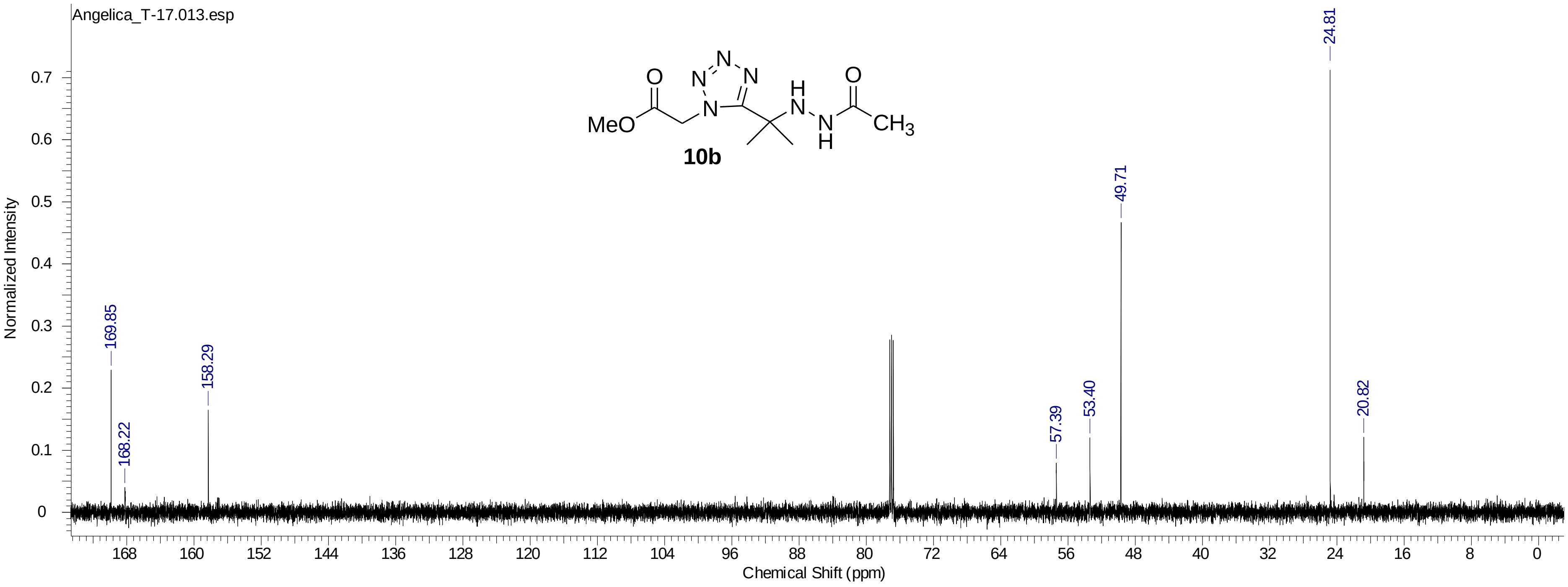

Figure S5: ^{13}C NMR (150 MHz, $CDCl_3$) spectrum of compound **10b**.

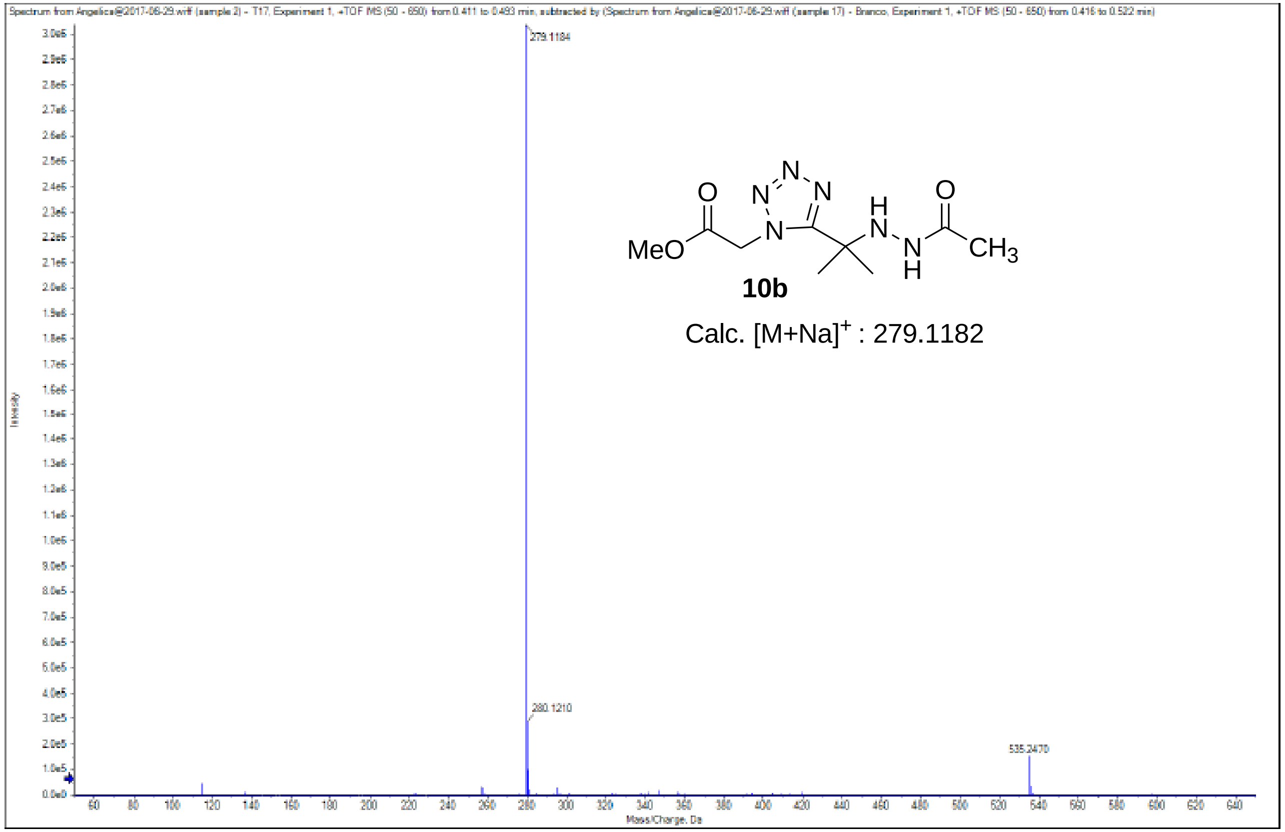

Figure S6: ESI-HRMS of compound **10b.**

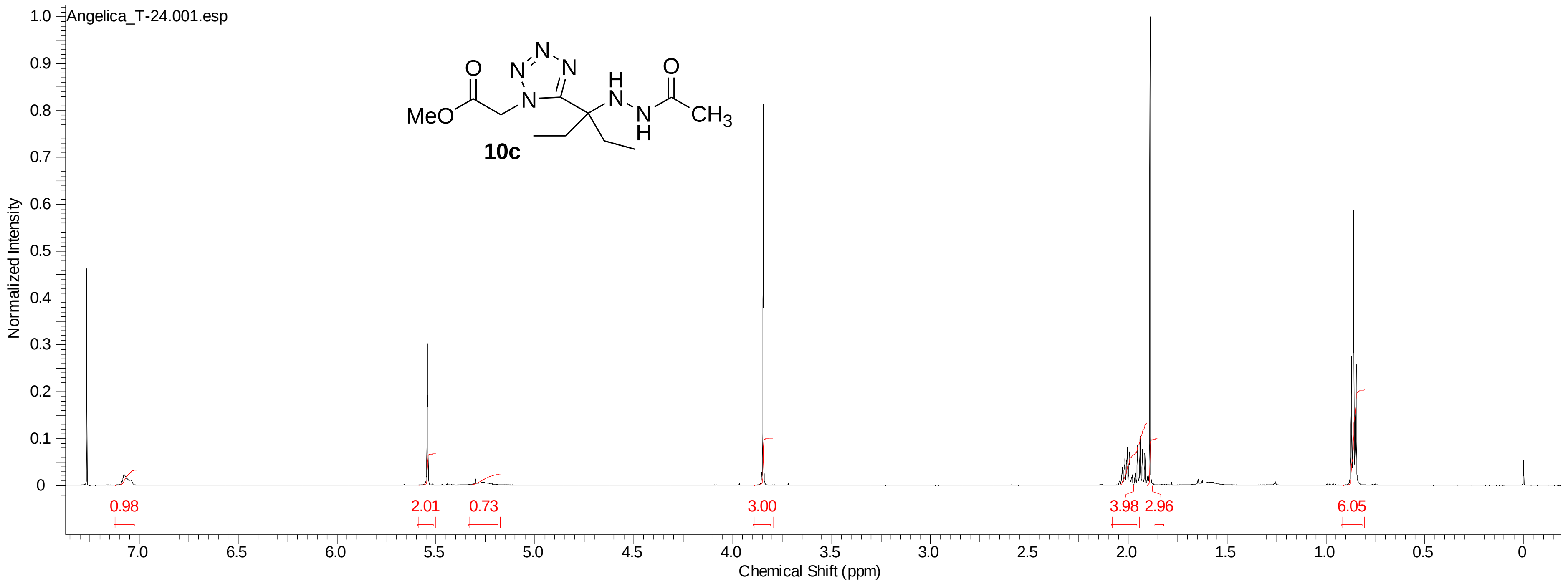

Figure S7: ^{1}H NMR (600 MHz, $CDCl_3$) spectrum of compound **10c**.

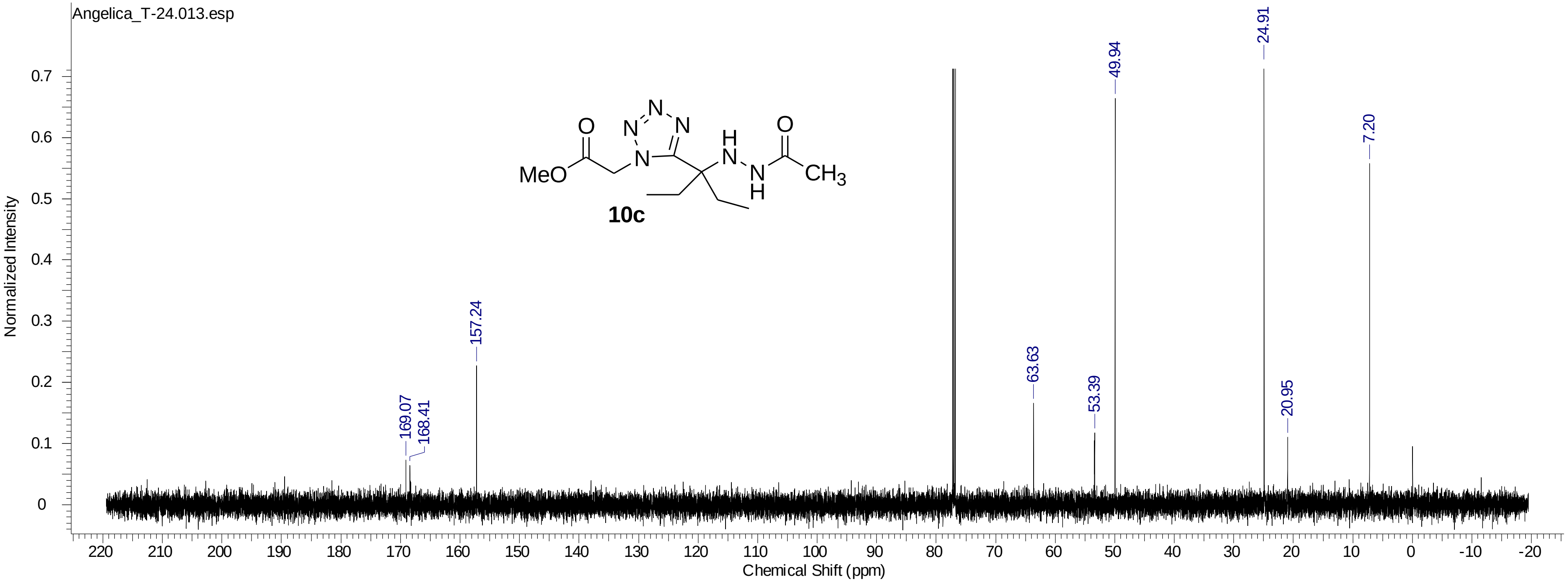

Figure S8: ^{13}C NMR (150 MHz, $CDCl_3$) spectrum of compound **10c**.

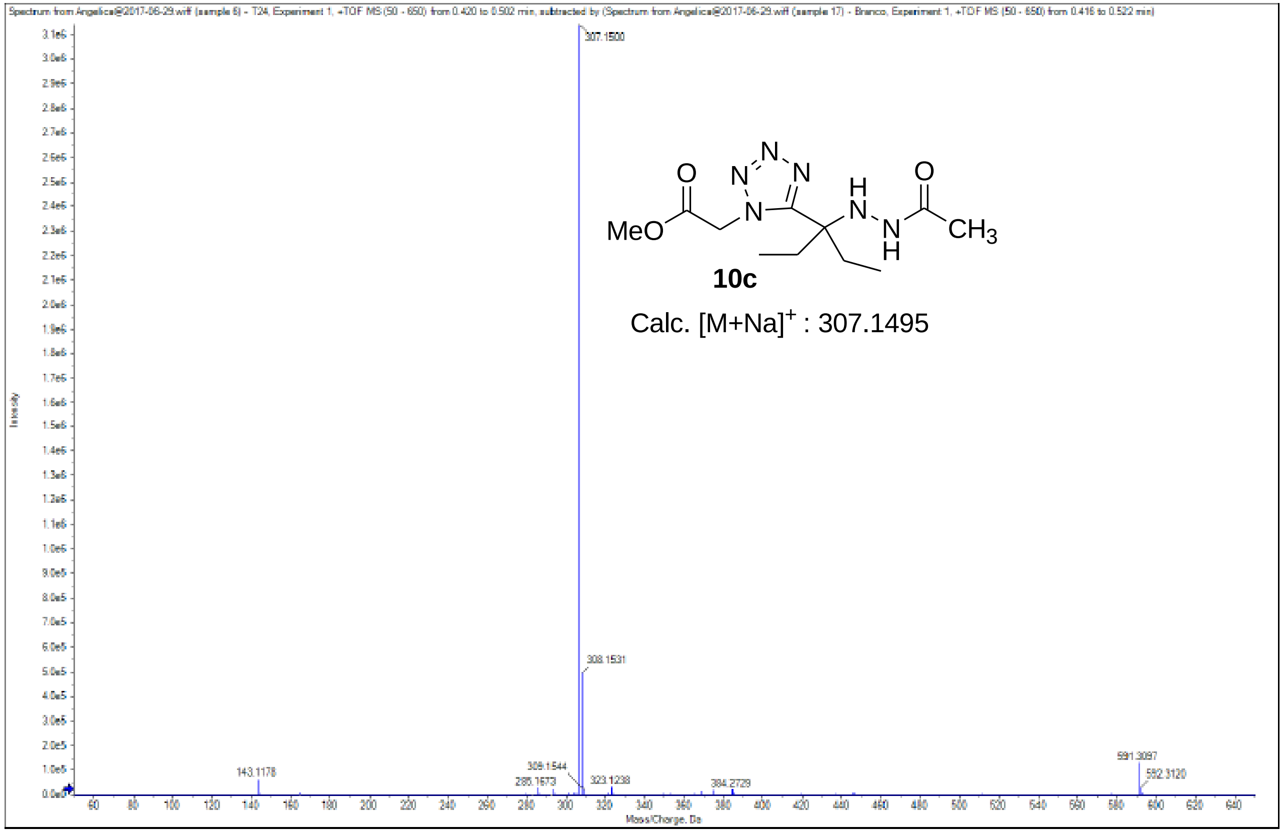

Figure S9: ESI-HRMS of compound **10c.**

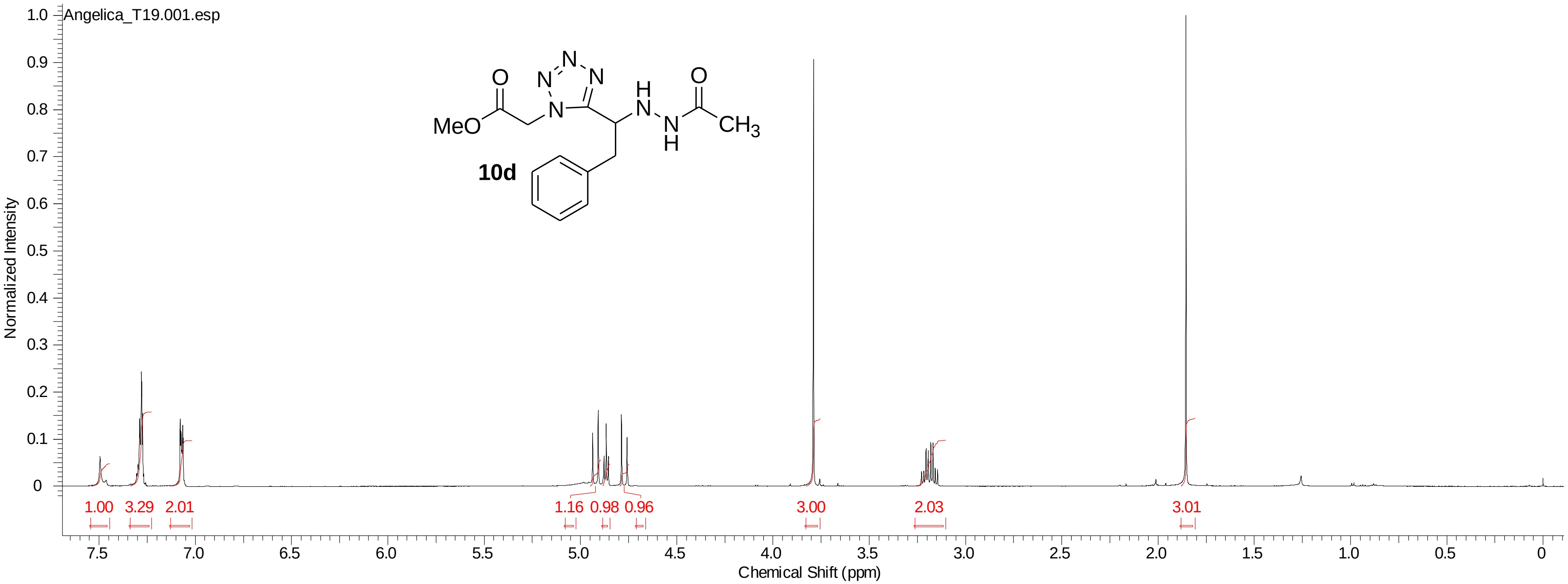

Figure S10: ^{1}H NMR (600 MHz, $CDCl_3$) spectrum of compound **10d**.

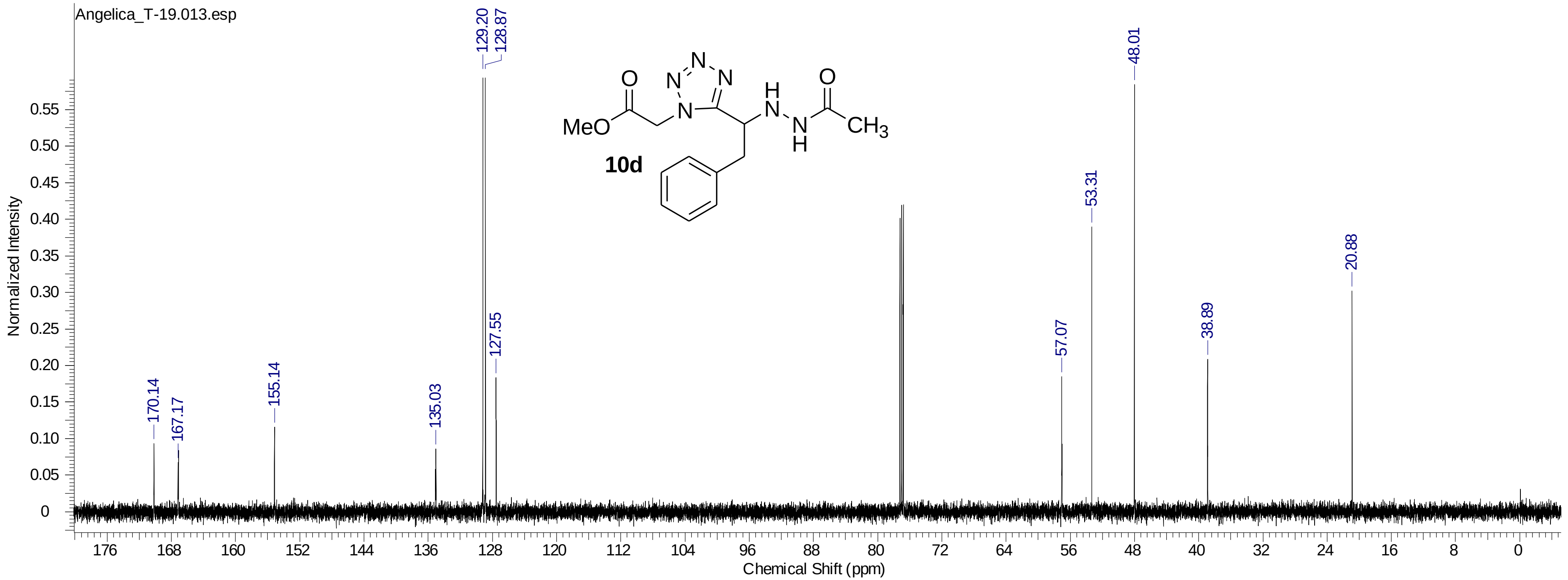

Figure S11: ^{13}C NMR (150 MHz, $CDCl_3$) spectrum of compound **10d**.

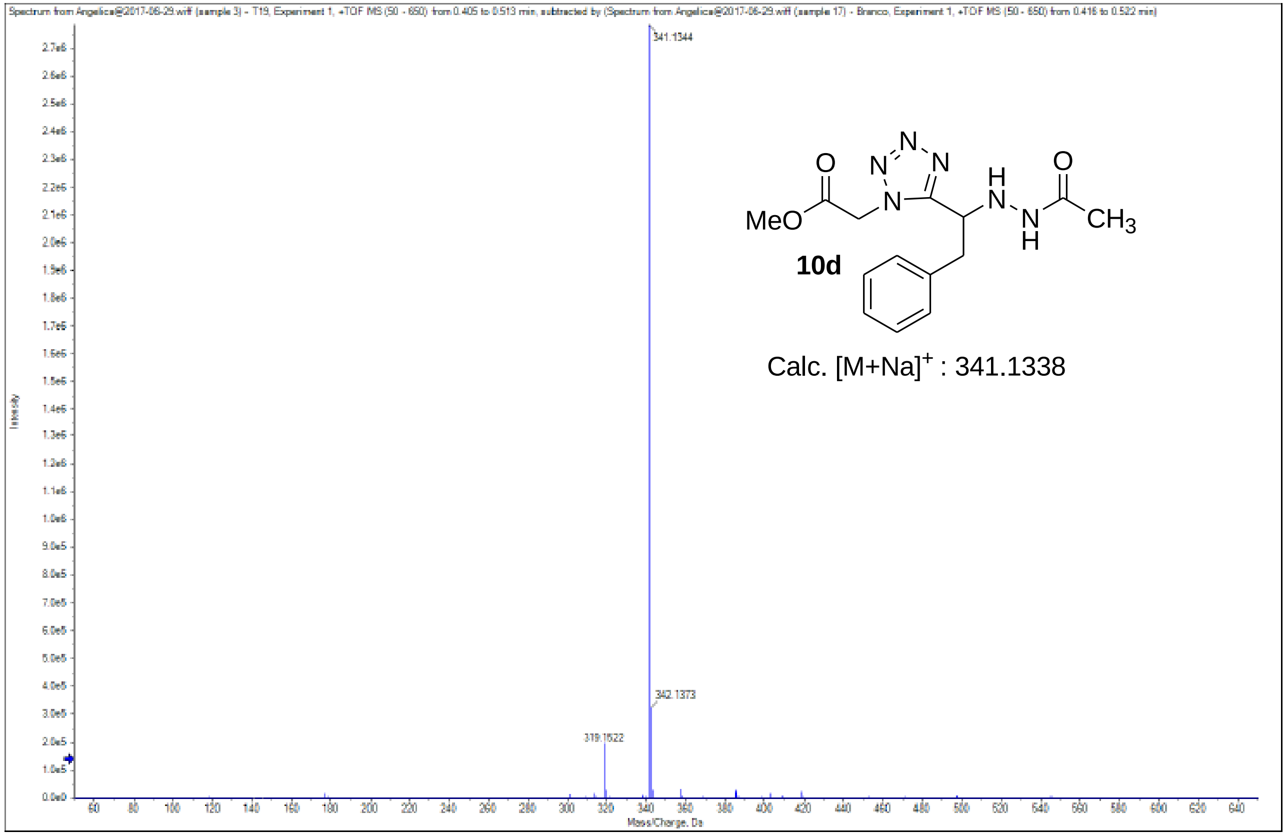

Figure S12: ESI-HRMS of compound **10d.**

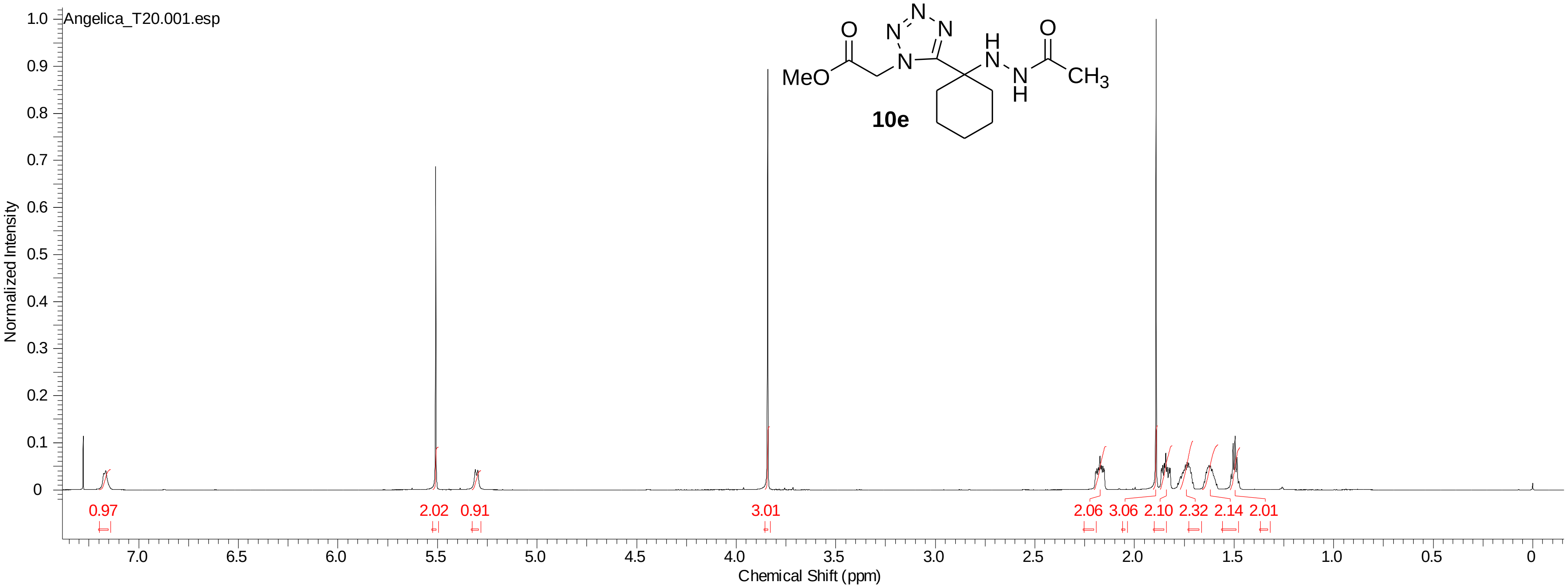

Figure S13: ^{1}H NMR (600 MHz, $CDCl_3$) spectrum of compound **10e**.

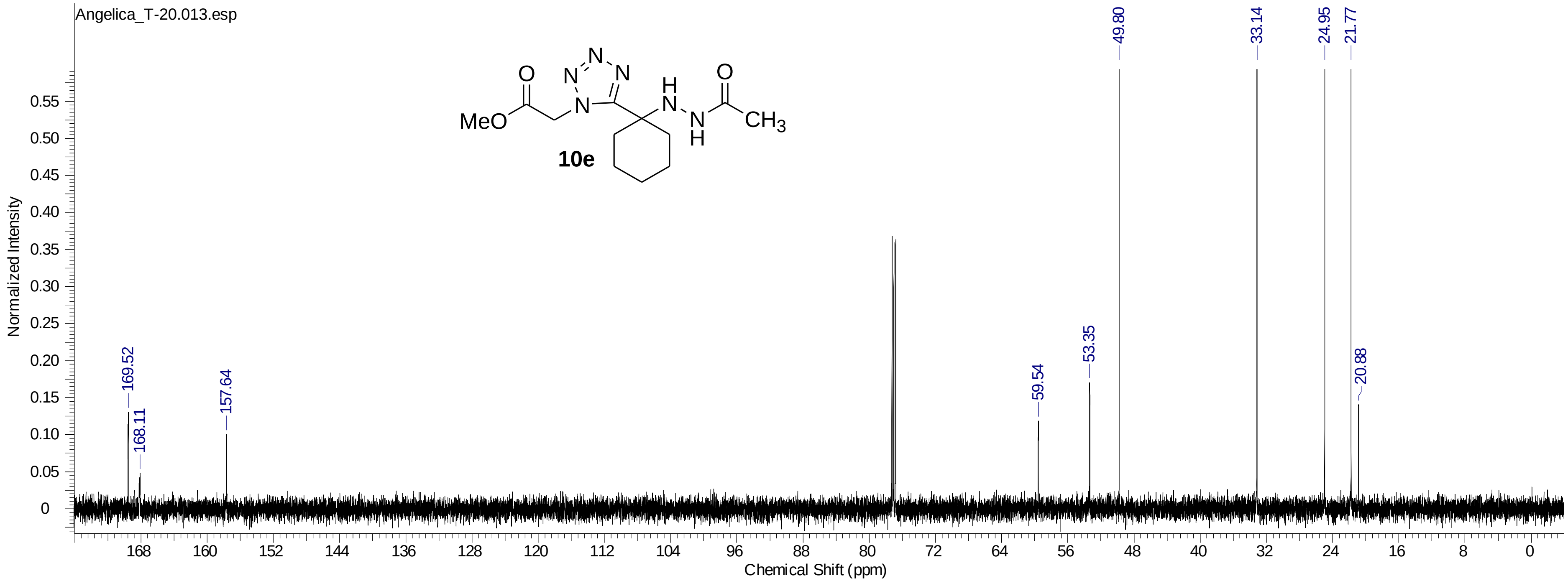

Figure S14: ^{13}C NMR (150 MHz, $CDCl_3$) spectrum of compound **10e**.

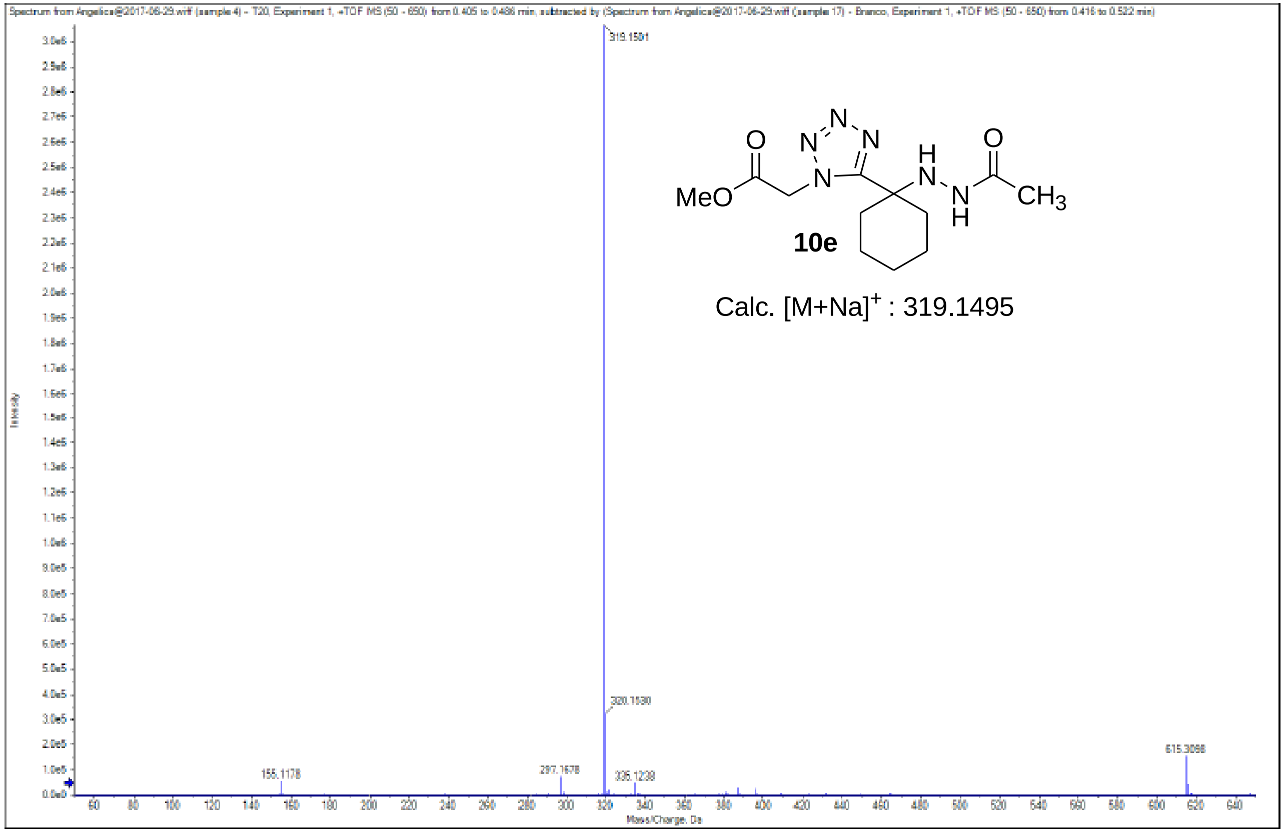

Figure S15: ESI-HRMS of compound **10e.**

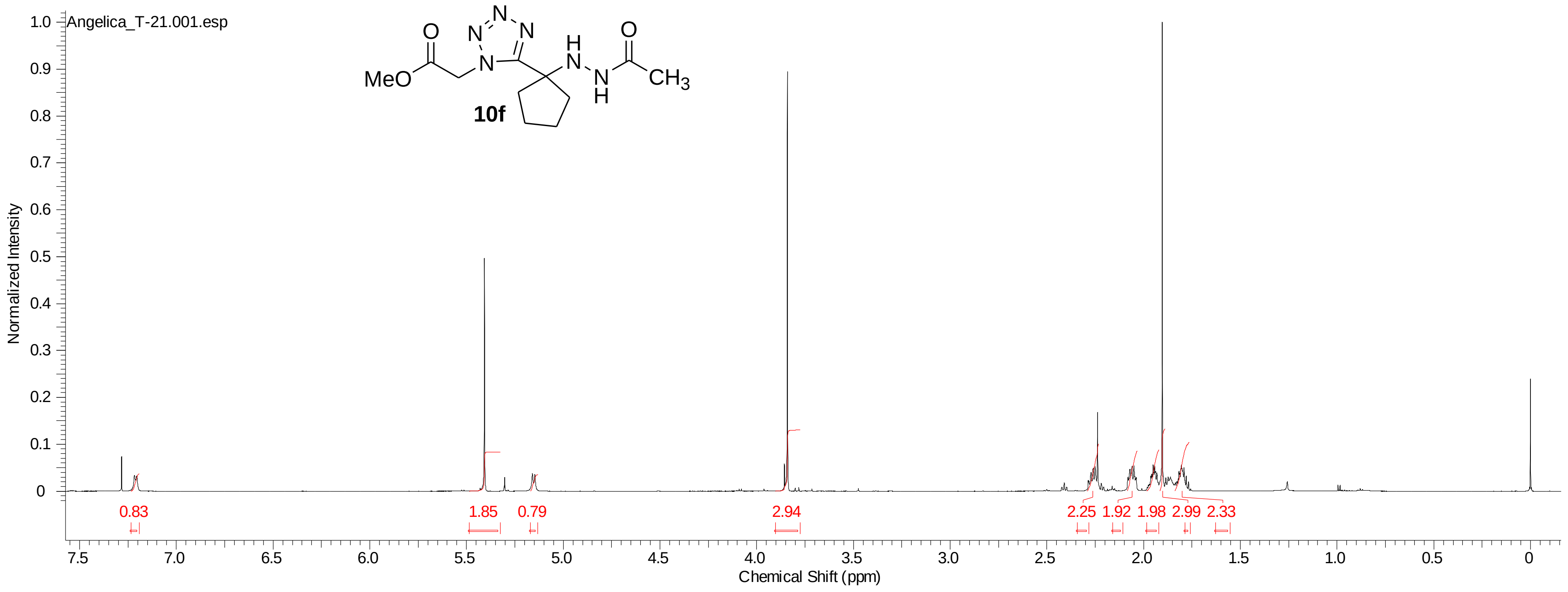

Figure S16: ^{1}H NMR (600 MHz, $CDCl_3$) spectrum of compound **10f**.

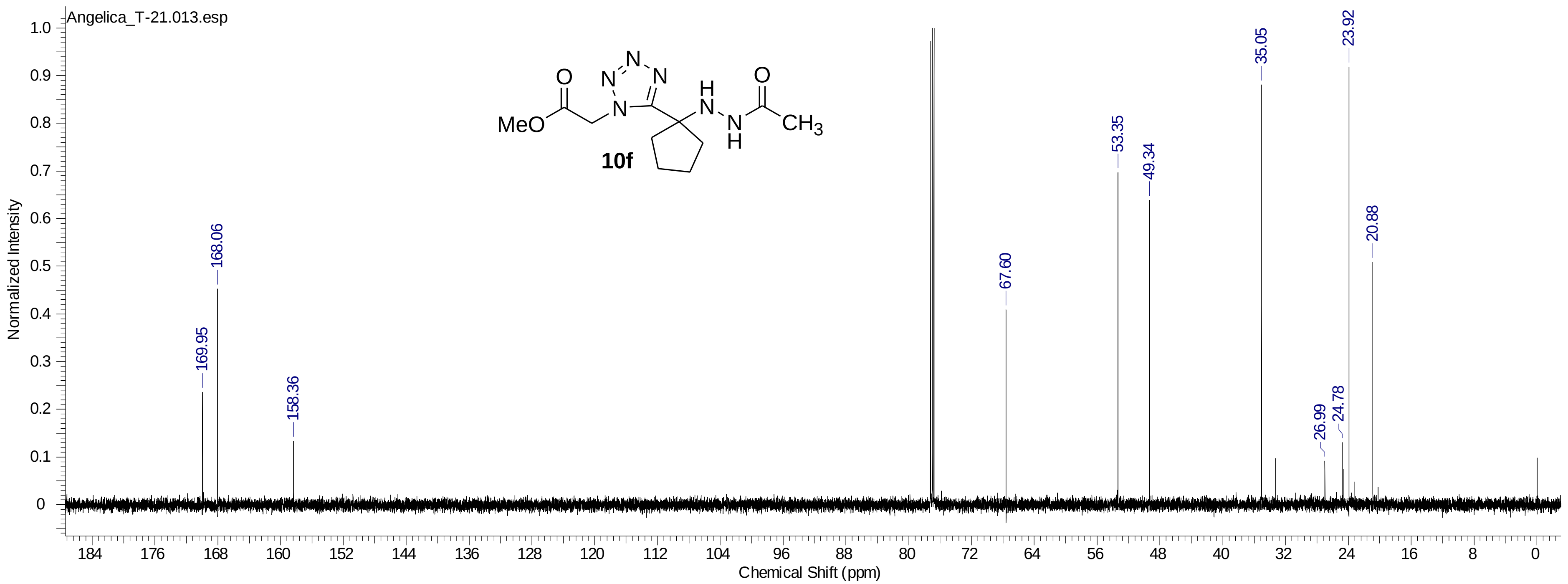

Figure S17: ^{13}C NMR (150 MHz, $CDCl_3$) spectrum of compound **10f**.

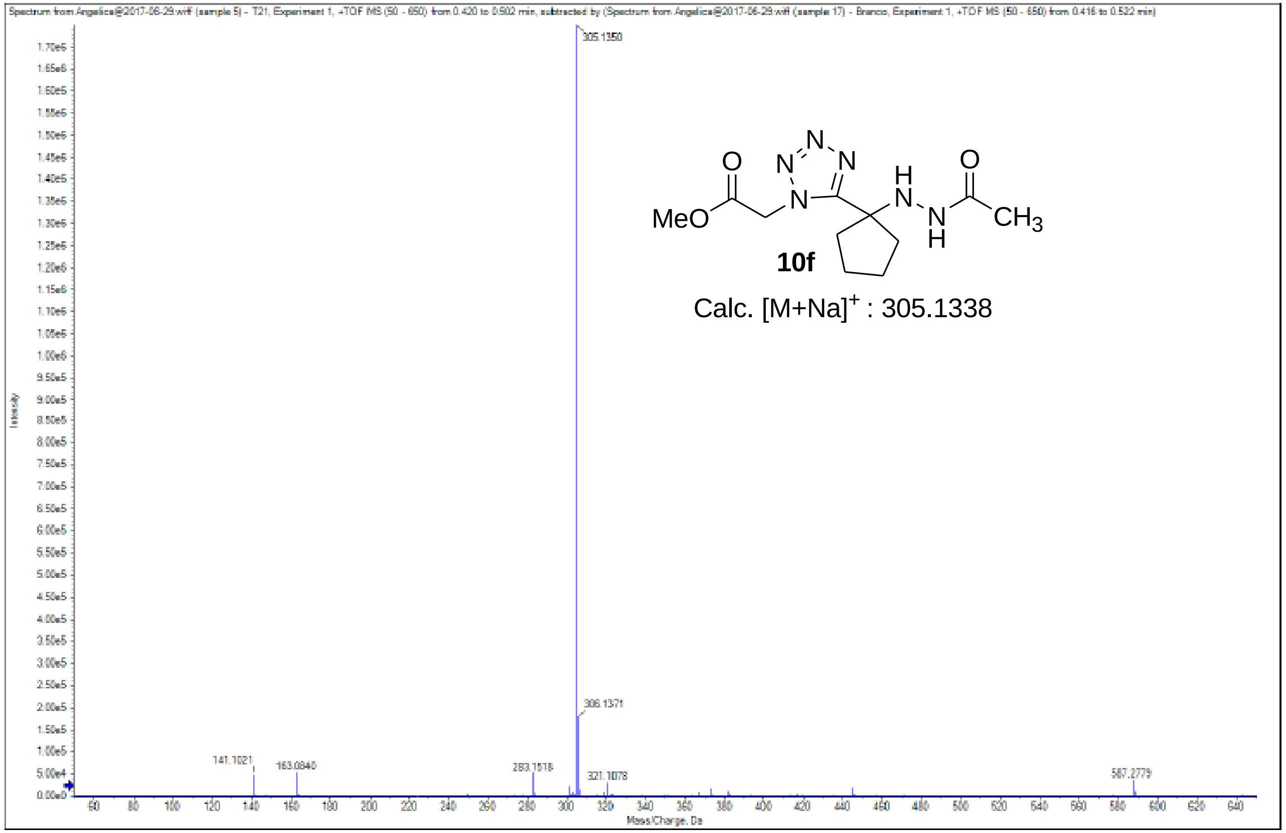

Figure S18: ESI-HRMS of compound **10f.**

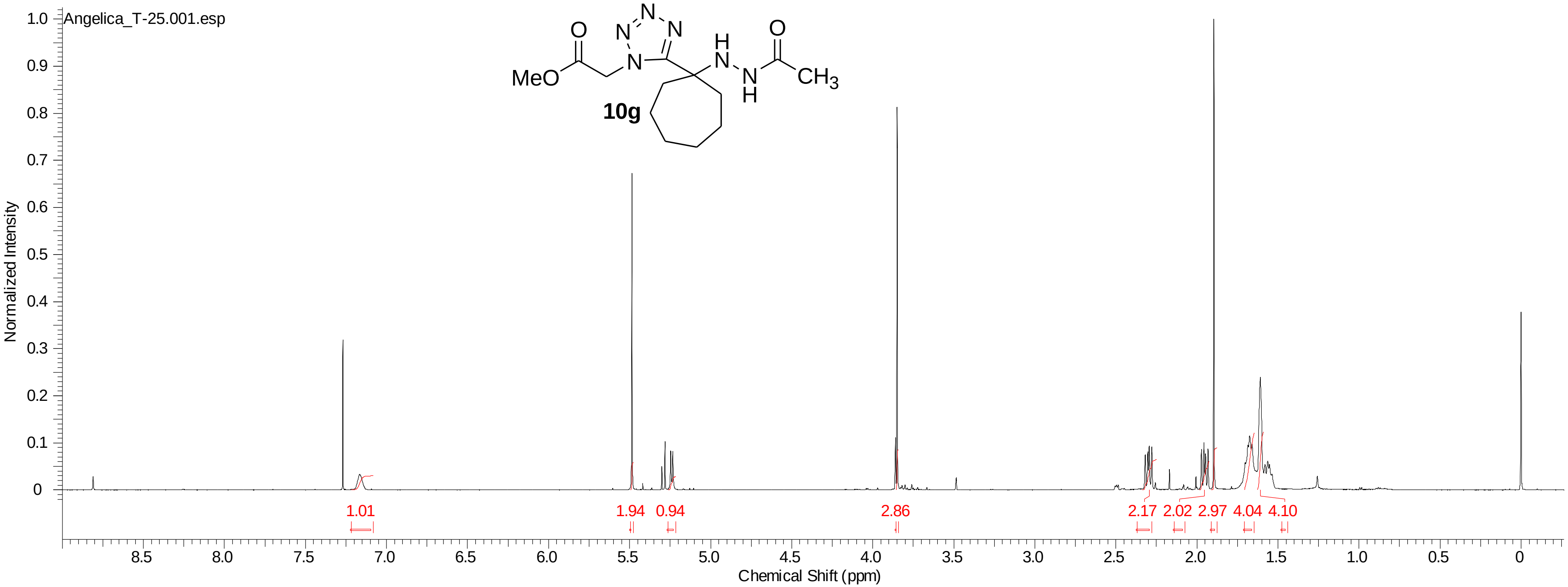

Figure S19: ^{1}H NMR (600 MHz, $CDCl_3$) spectrum of compound **10g**.

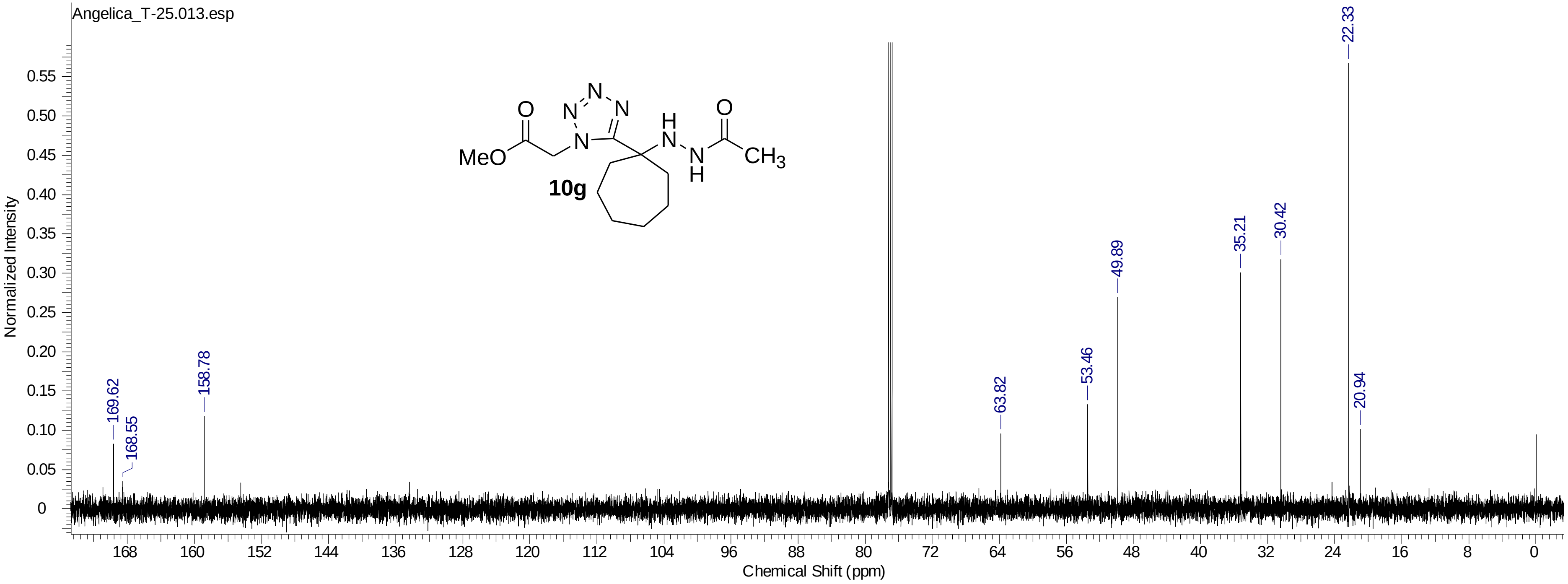

Figure S20: ^{13}C NMR (150 MHz, $CDCl_3$) spectrum of compound **10g**.

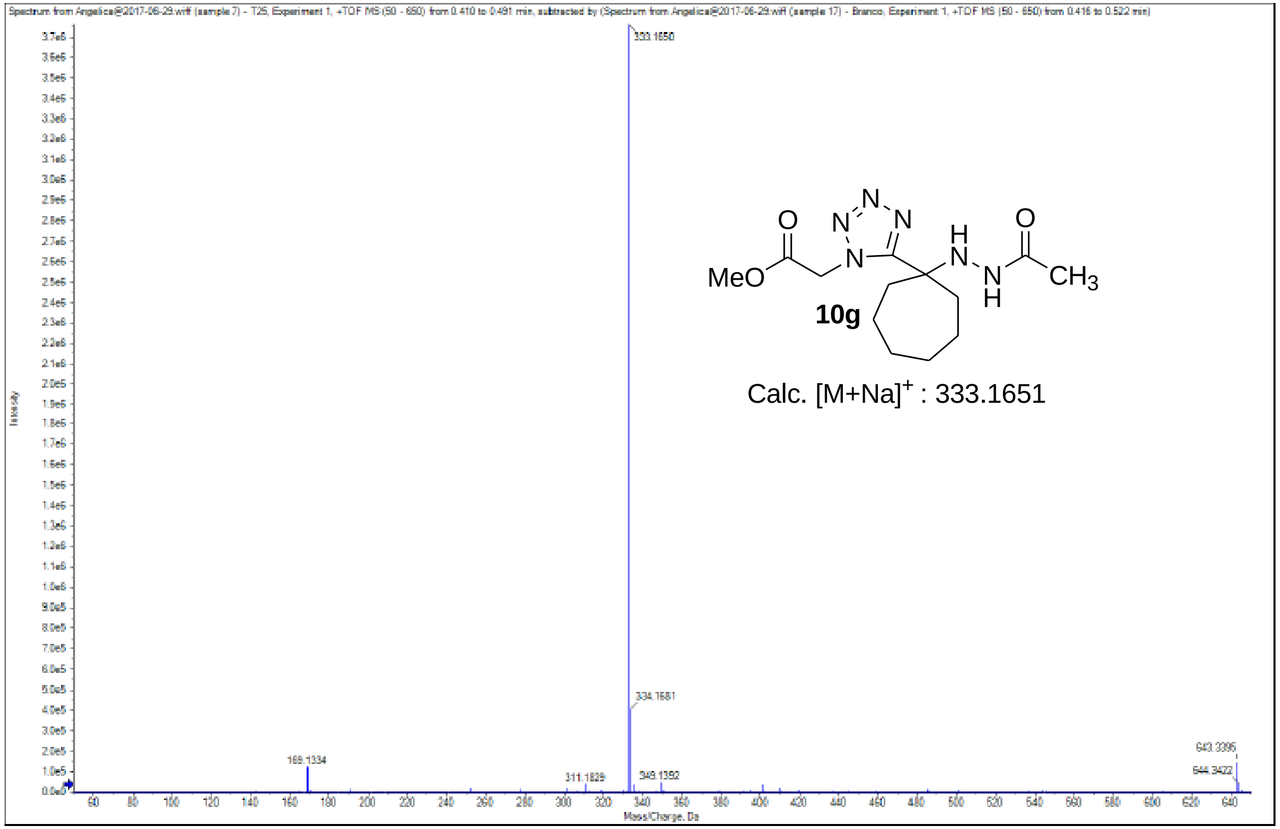

Figure S21: ESI-HRMS of compound **10g.**

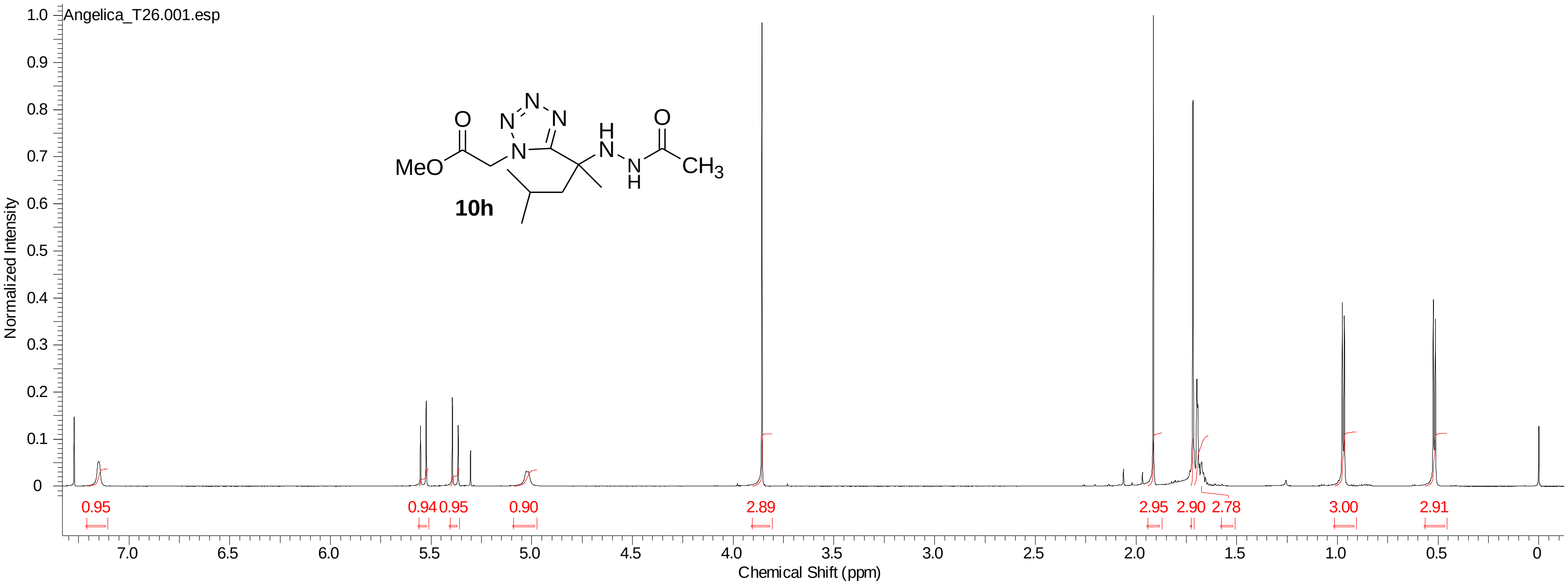

Figure S22: ^{1}H NMR (600 MHz, $CDCl_3$) spectrum of compound **10h**.

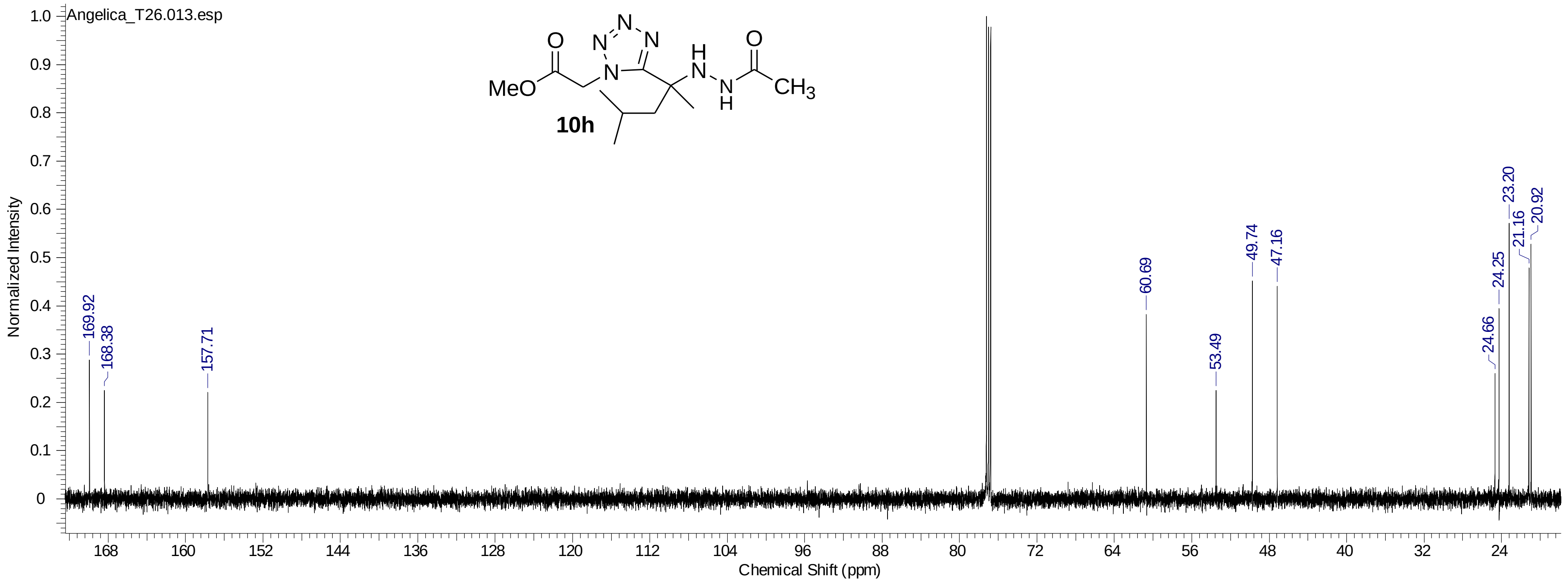

Figure S23: ^{13}C NMR (150 MHz, $CDCl_3$) spectrum of compound **10h**.

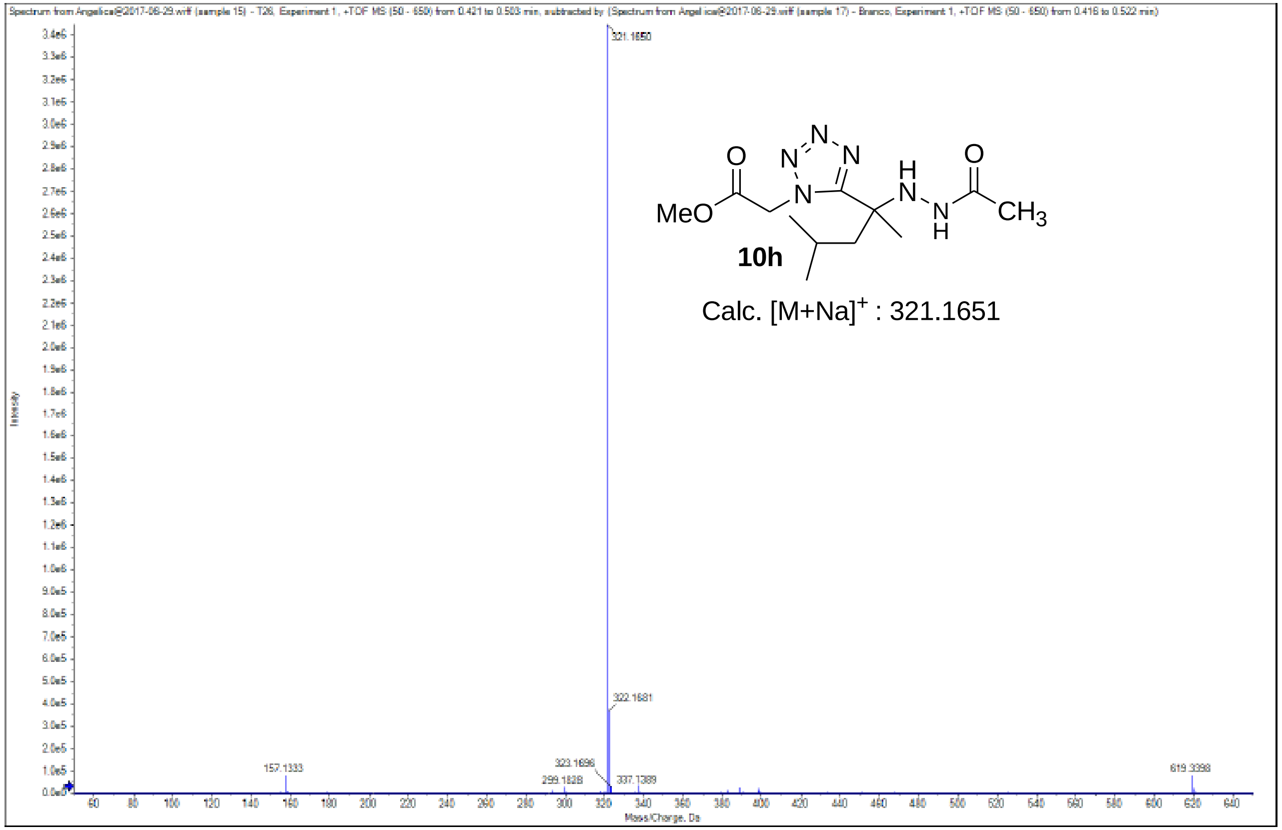

Figure S24: ESI-HRMS of compound **10h.**

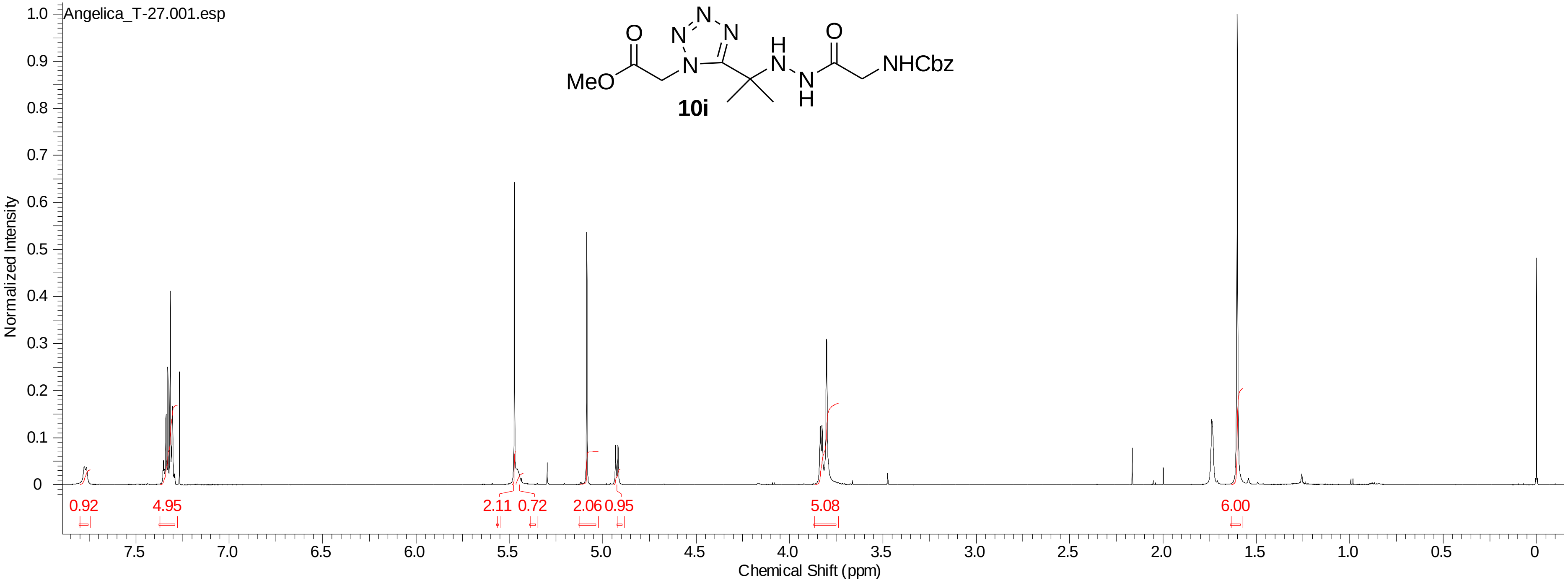

Figure S25: ^{1}H NMR (600 MHz, $CDCl_3$) spectrum of compound **10i**.

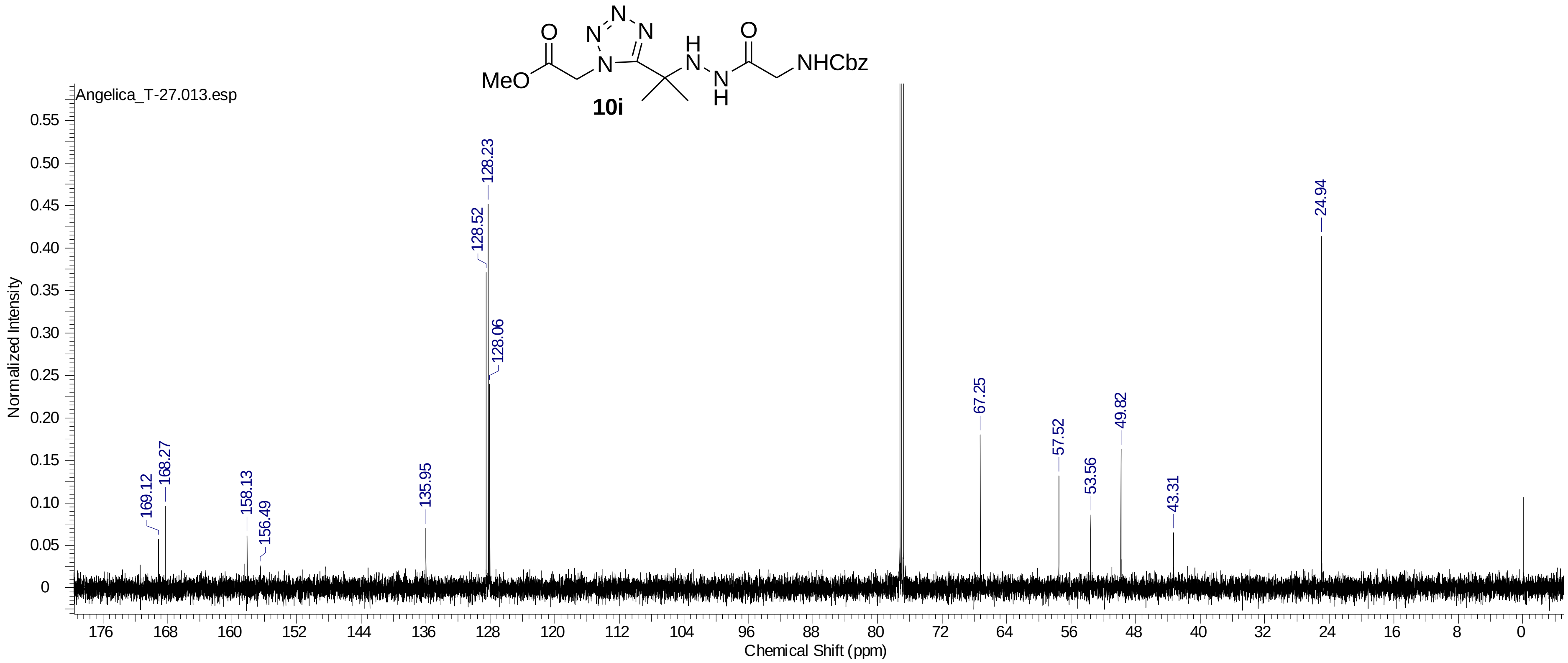

Figure S26: ^{13}C NMR (150 MHz, $CDCl_3$) spectrum of compound **10i**.

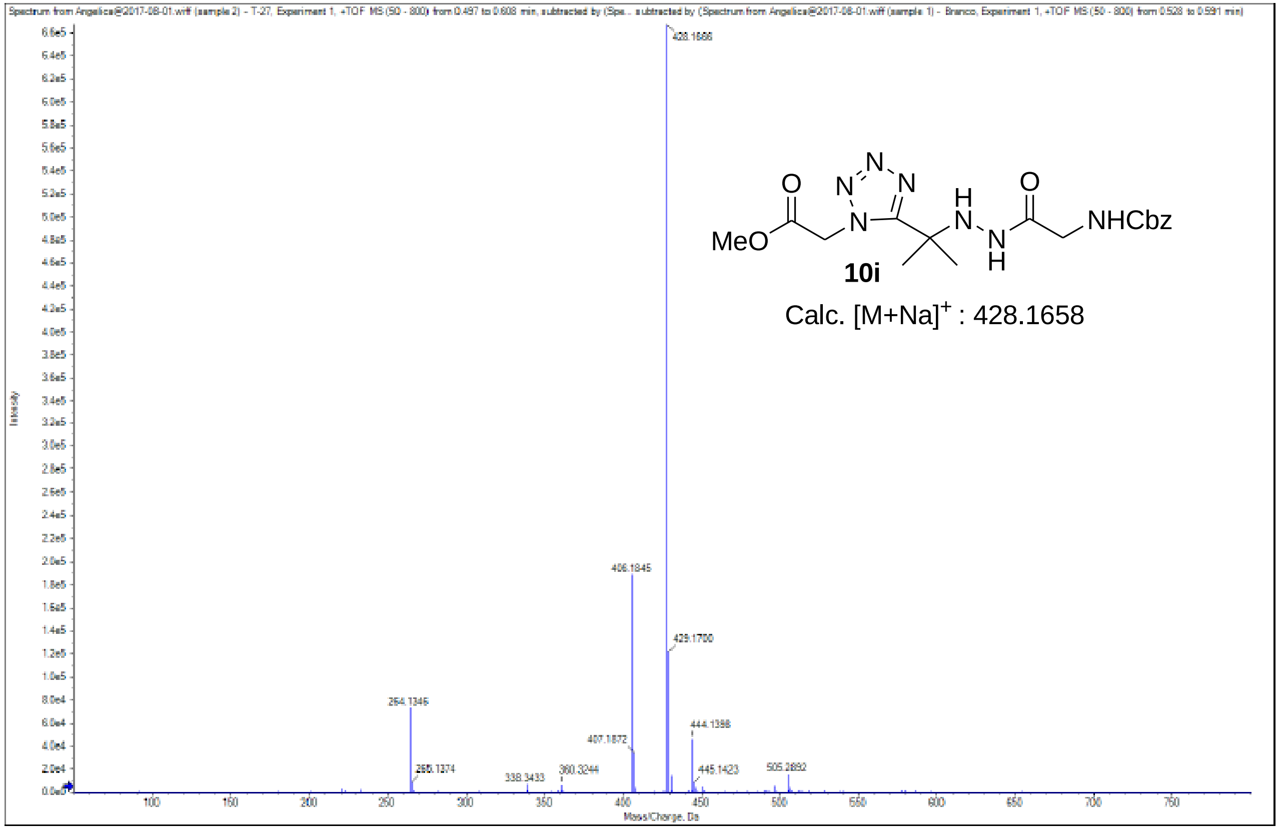

Figure S27: ESI-HRMS of compound **10i.**

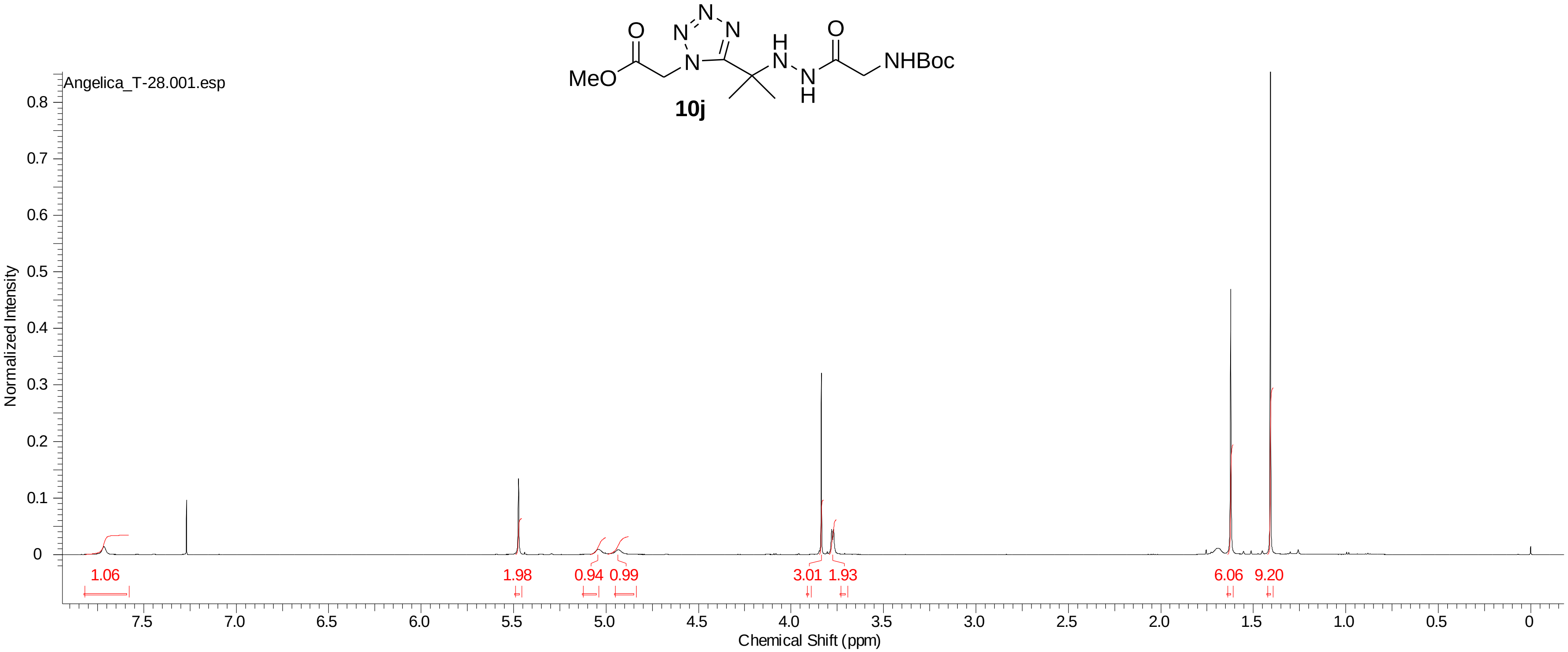

Figure S28: ^{1}H NMR (600 MHz, $CDCl_3$) spectrum of compound **10j**.

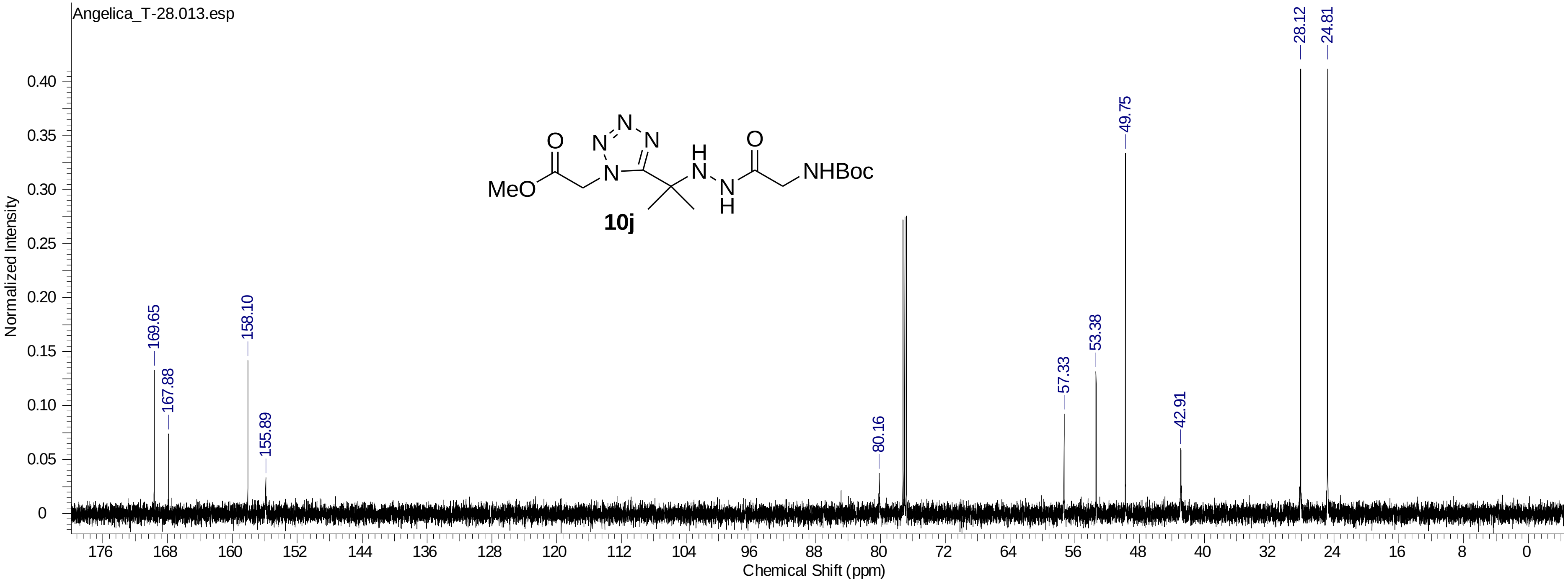

Figure S29: ^{13}C NMR (150 MHz, $CDCl_3$) spectrum of compound **10j**.

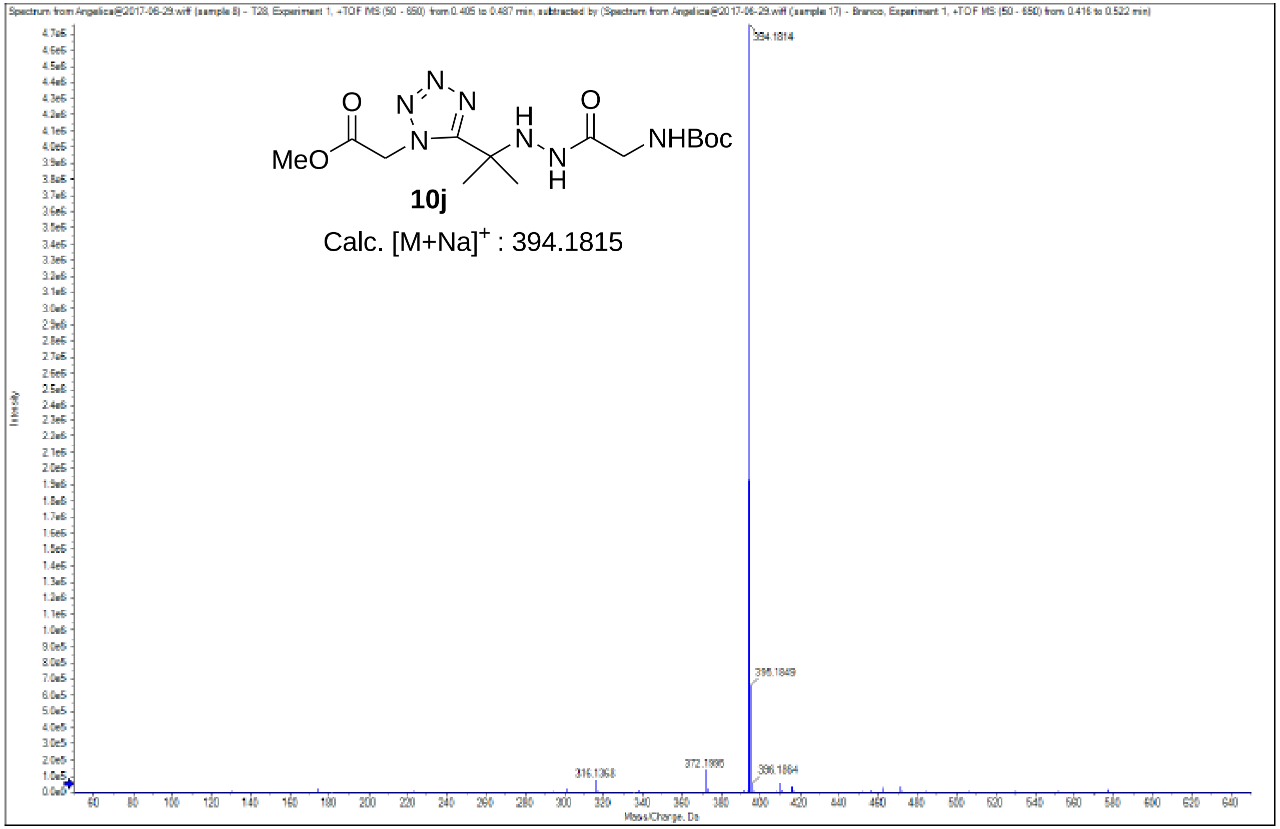

Figure S30: ESI-HRMS of compound **10j.**

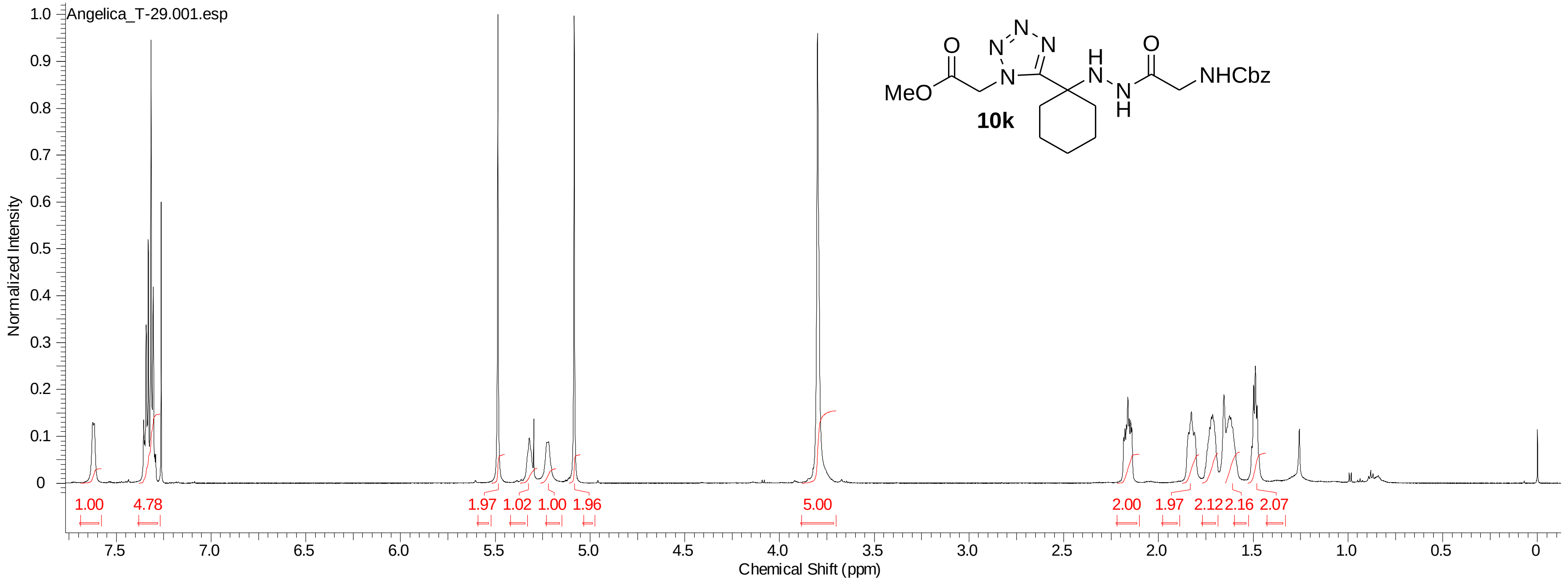

Figure S31: ^{1}H NMR (600 MHz, $CDCl_3$) spectrum of compound **10k**.

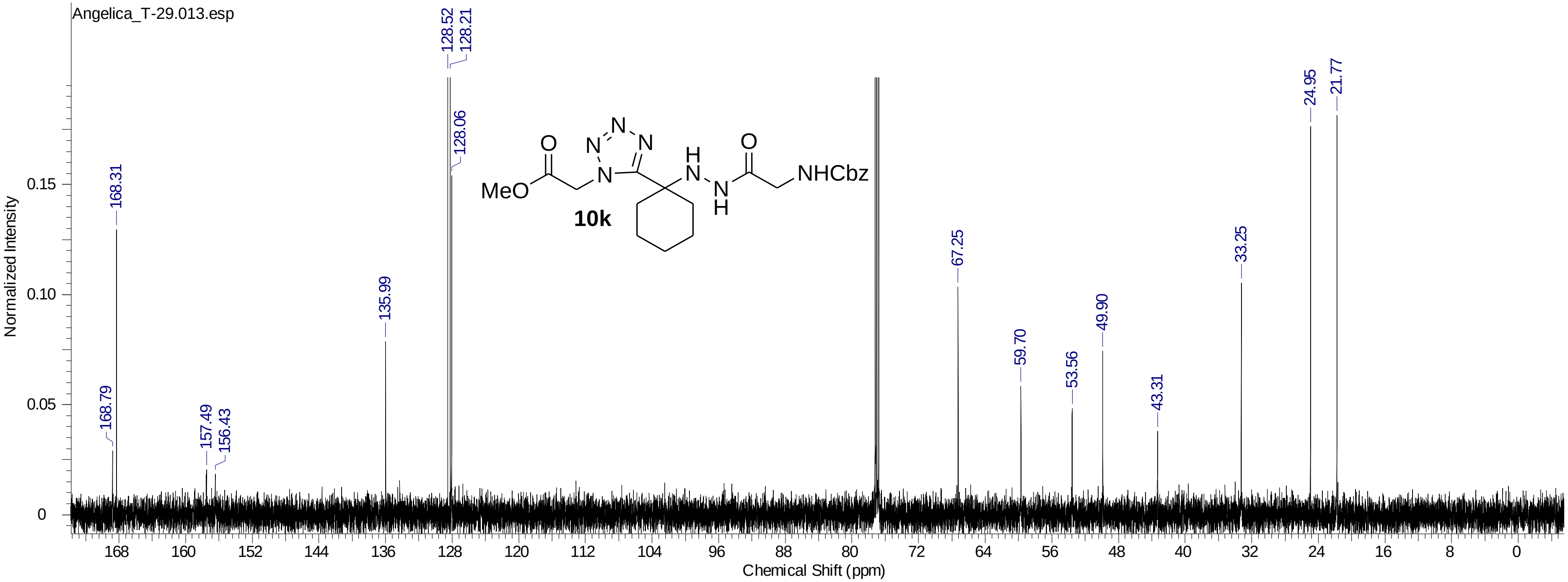

Figure S32: ^{13}C NMR (150 MHz, $CDCl_3$) spectrum of compound **10k**.

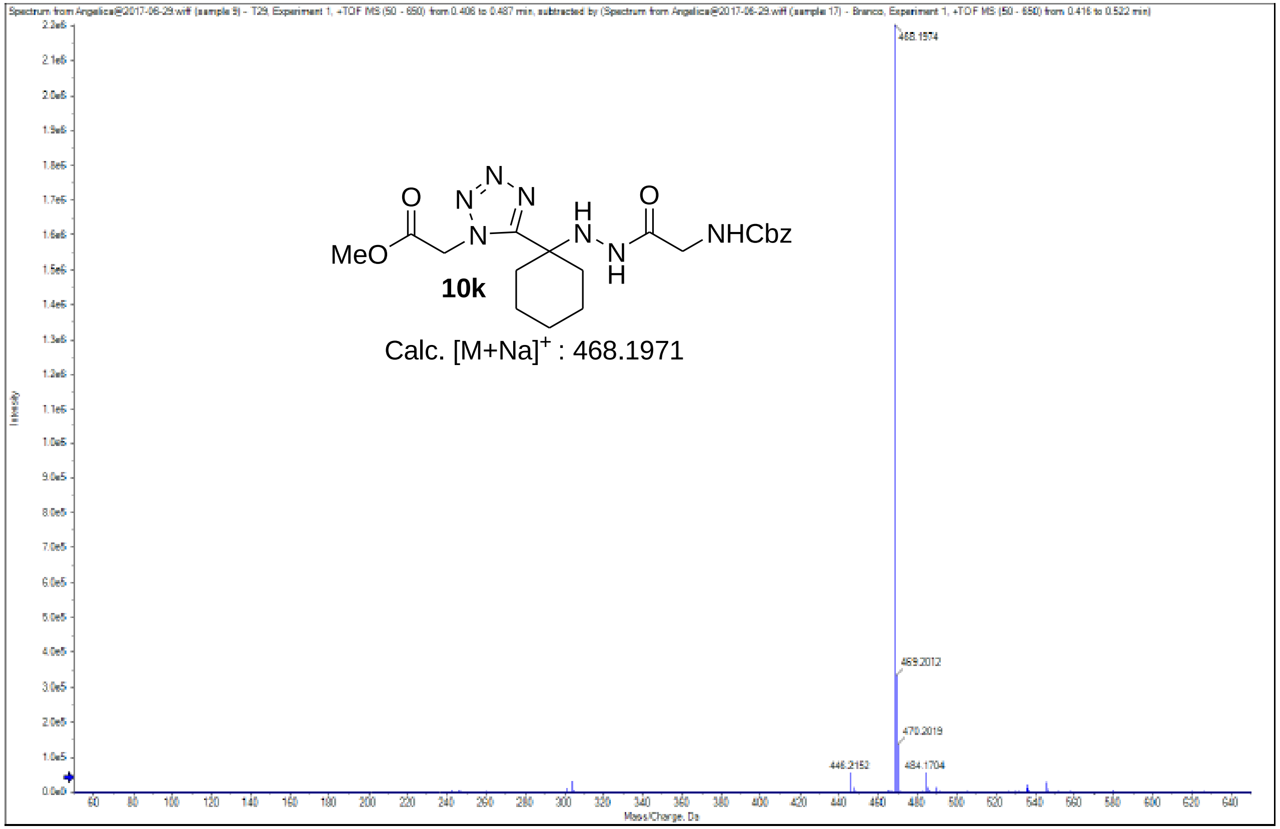

Figure S33: ESI-HRMS of compound **10k.**

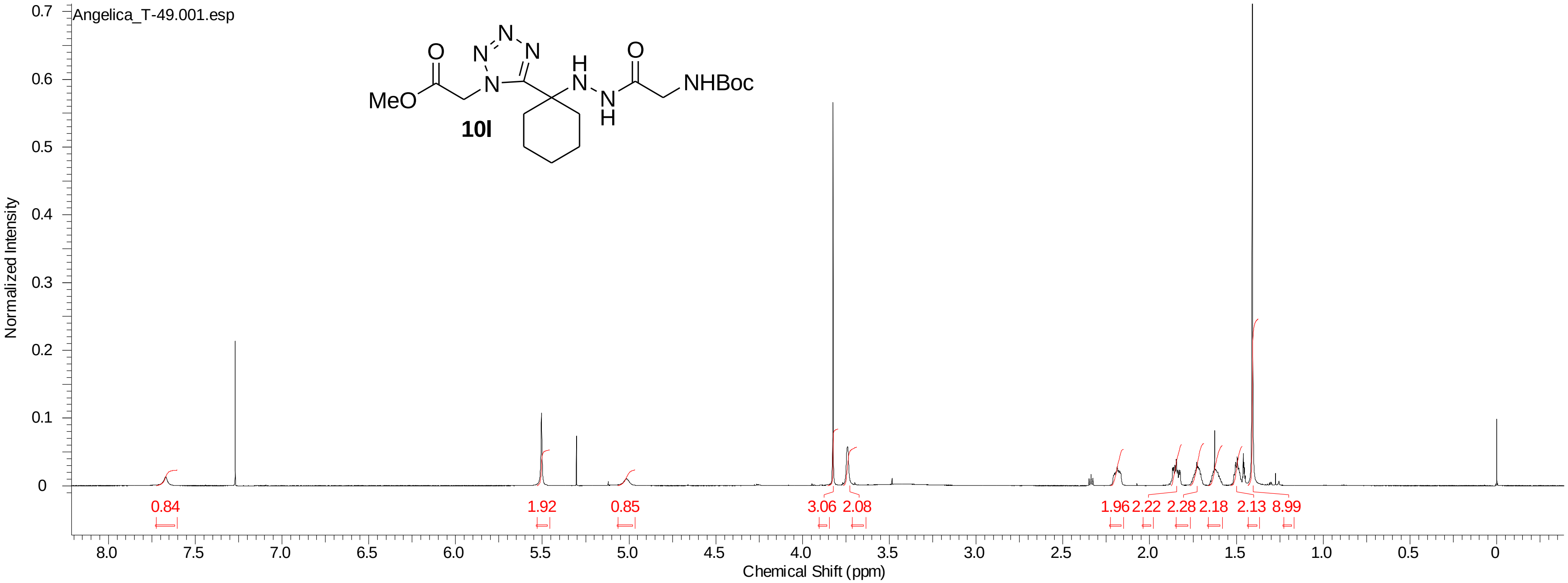

Figure S34: ^{1}H NMR (600 MHz, $CDCl_3$) spectrum of compound **10l**.

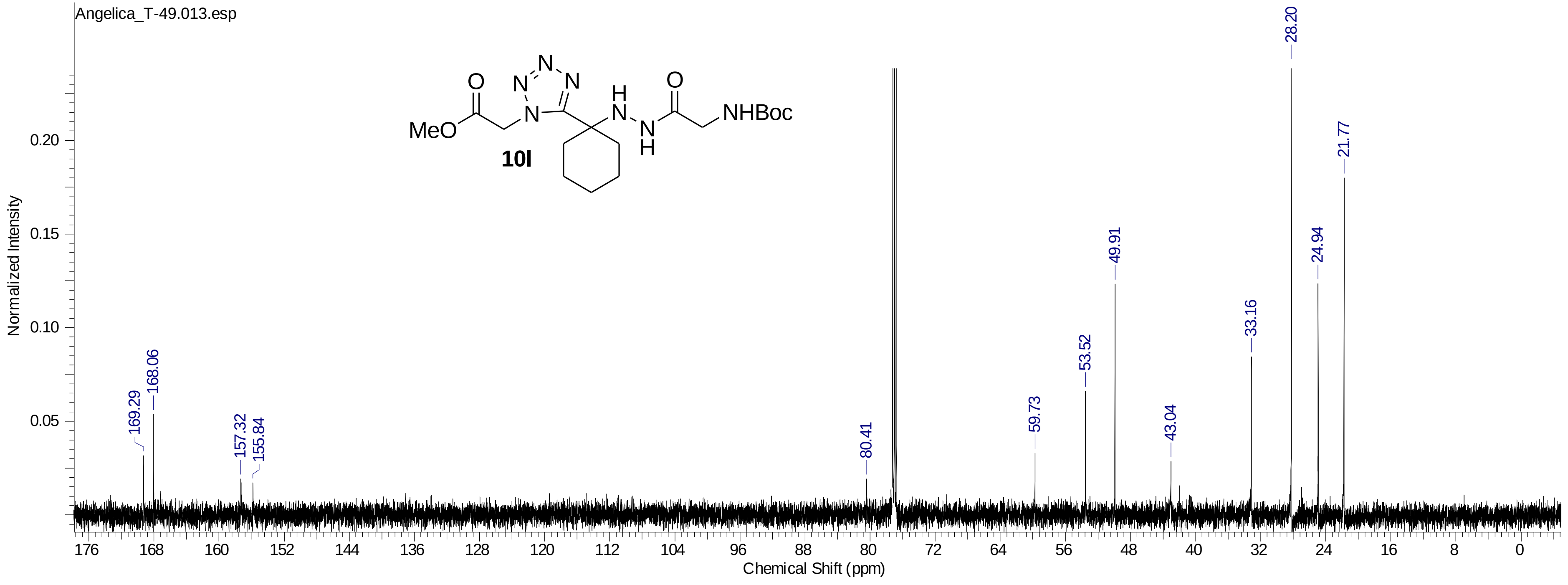

Figure S35: ^{13}C NMR (150 MHz, $CDCl_3$) spectrum of compound **10l**.

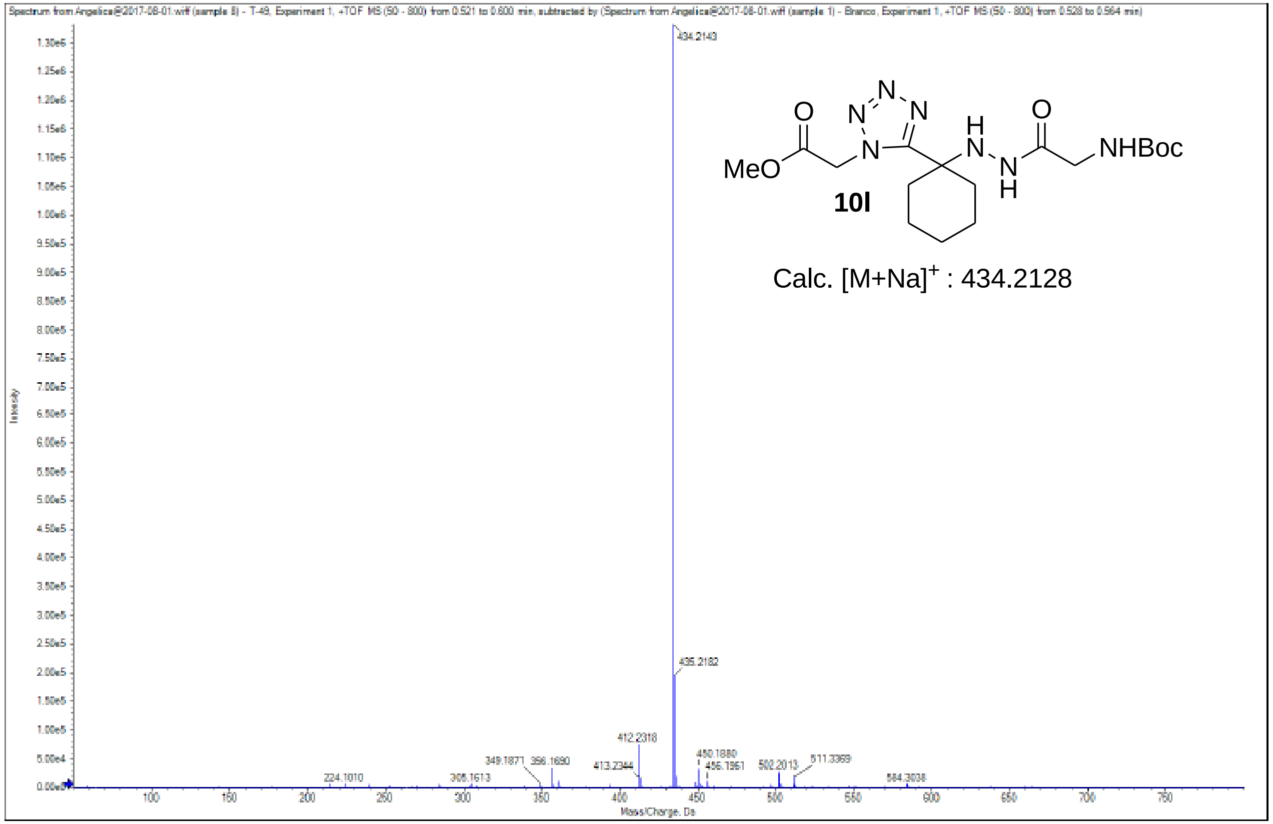

Figure S36: ESI-HRMS of compound **10l.**

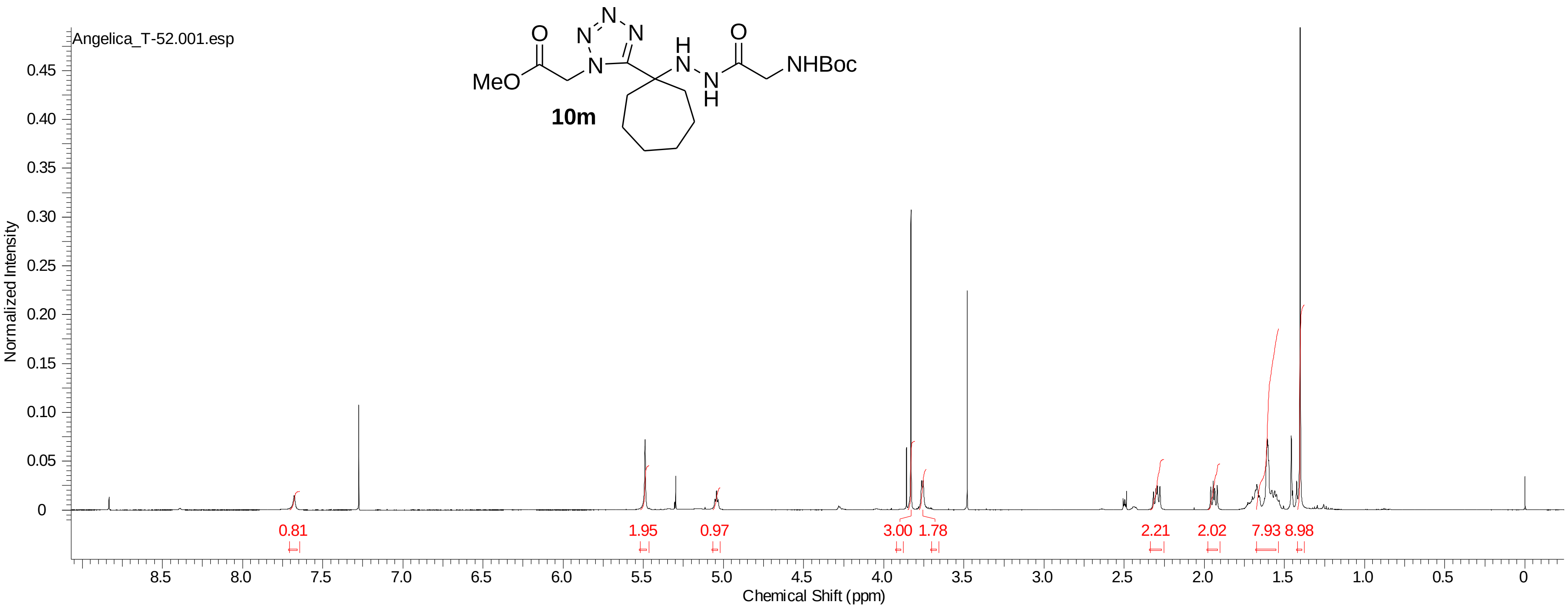

Figure S37: ^{1}H NMR (600 MHz, $CDCl_3$) spectrum of compound **10m**.

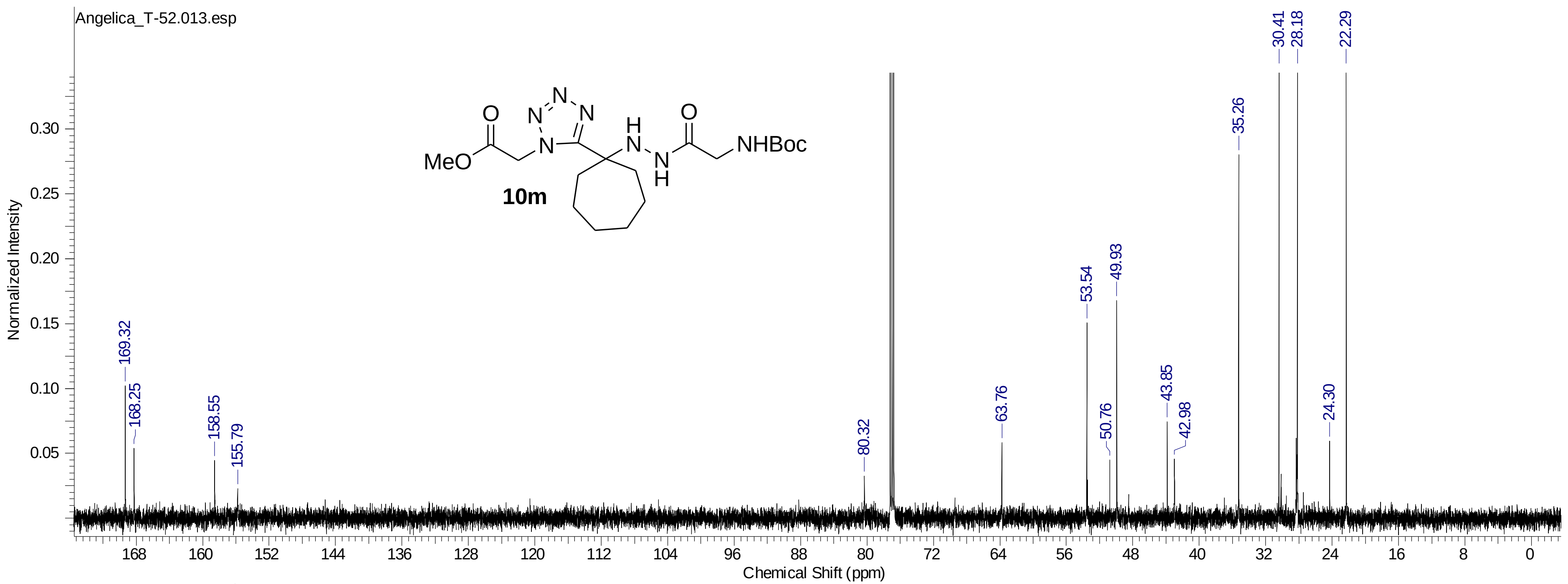

Figure S38: ^{13}C NMR (150 MHz, $CDCl_3$) spectrum of compound **10m**.

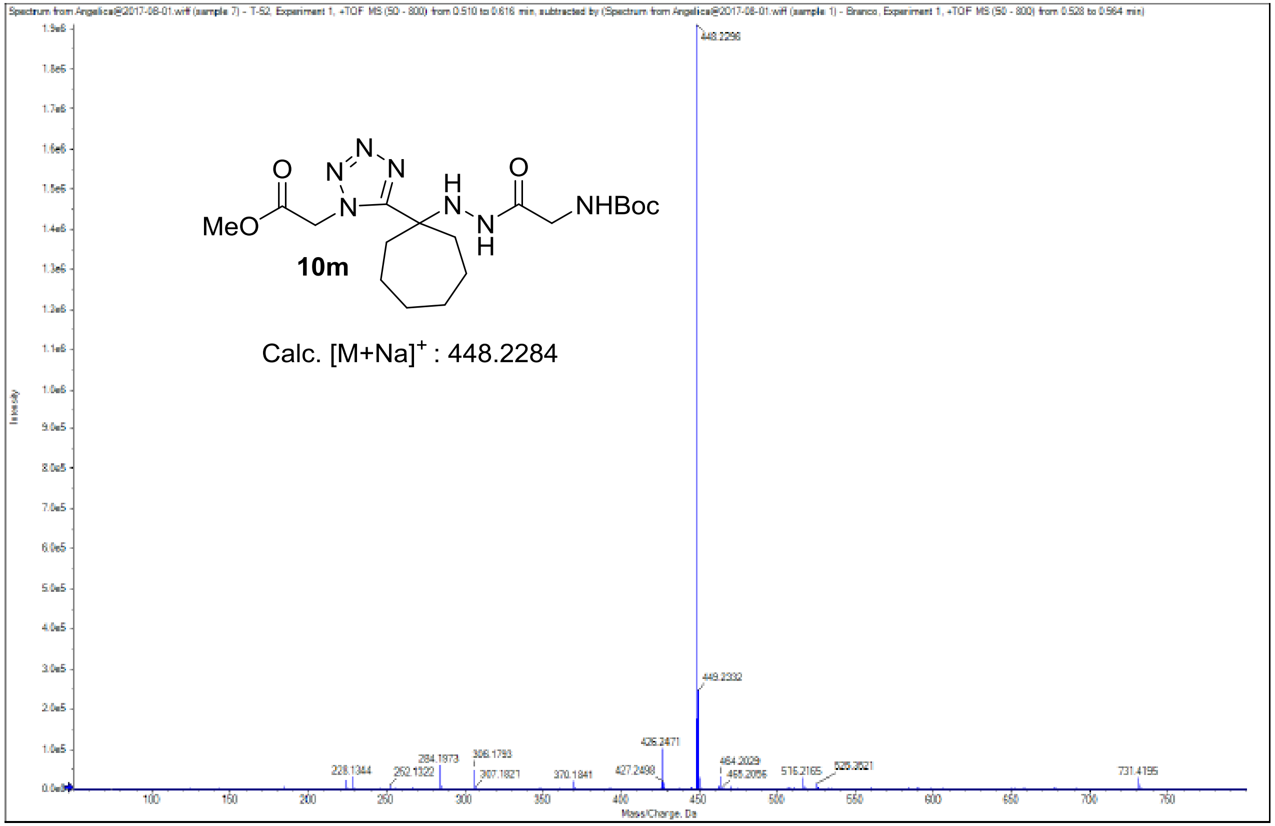

Figure S39: ESI-HRMS of compound **10m.**

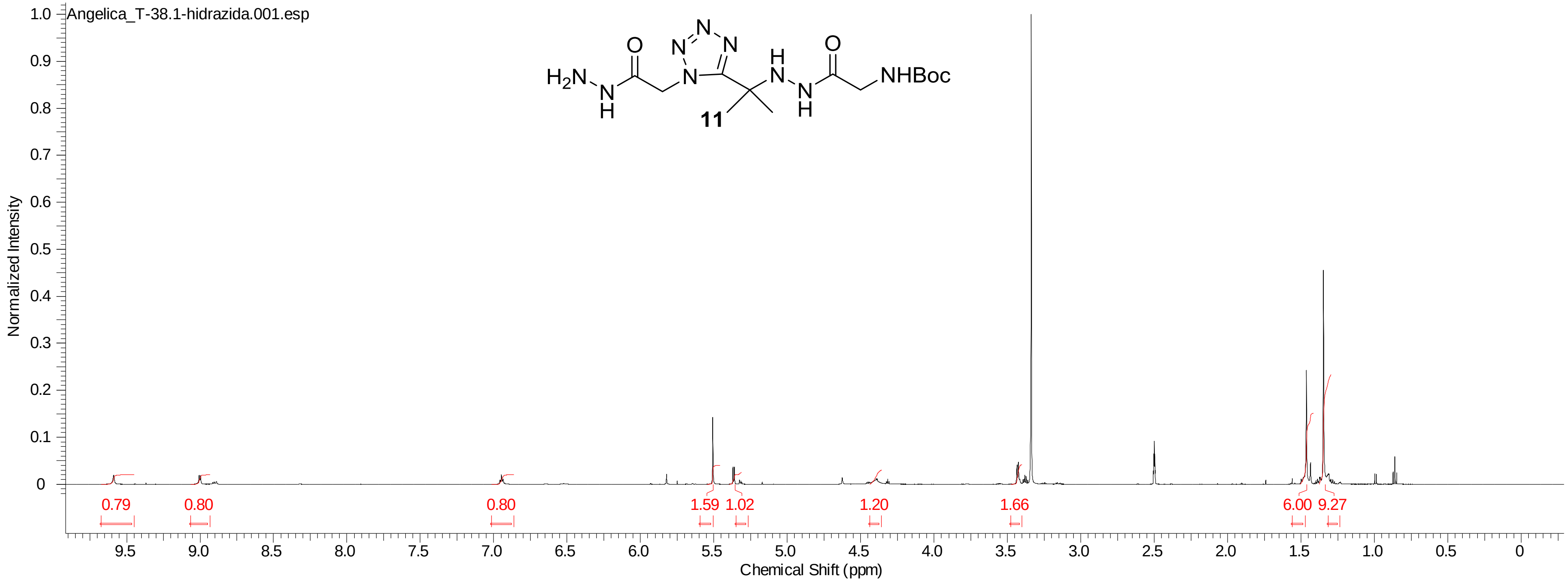

Figure S40: ^{1}H NMR (600 MHz, DMSO-d_6) spectrum of compound **11**.

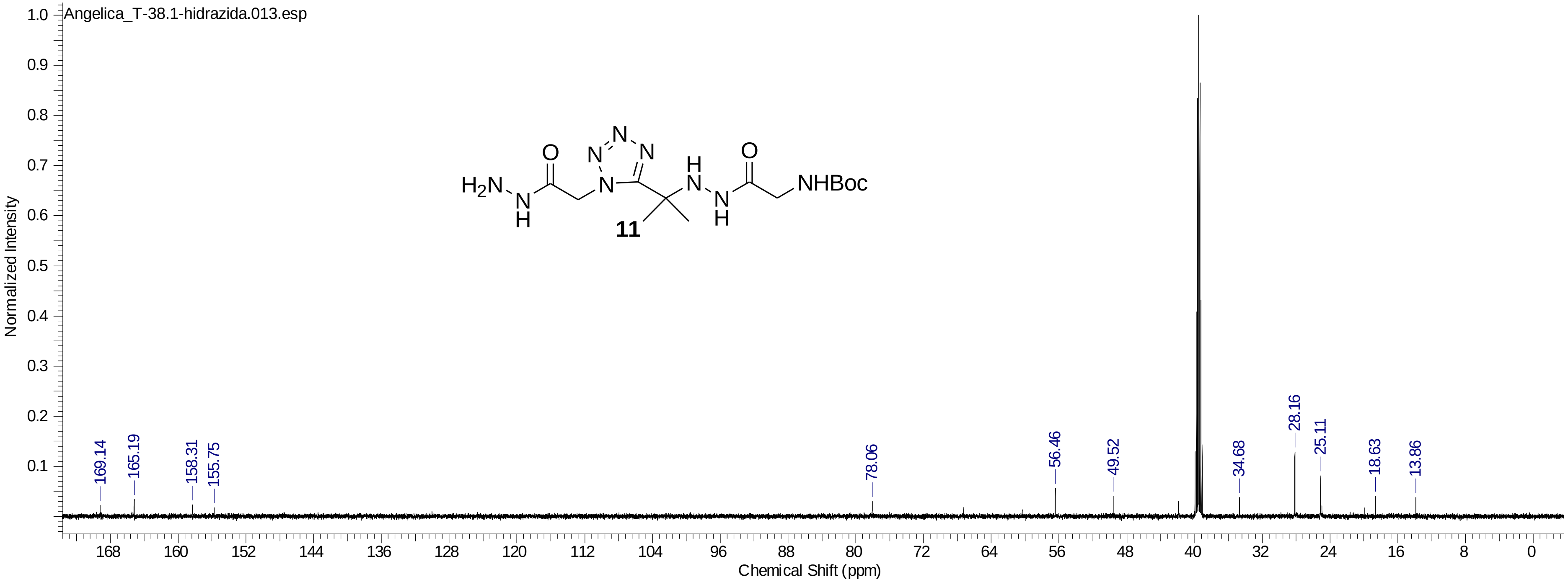

Figure S41: ^{13}C NMR (150 MHz, DMSO-d_6) spectrum of compound **11**.

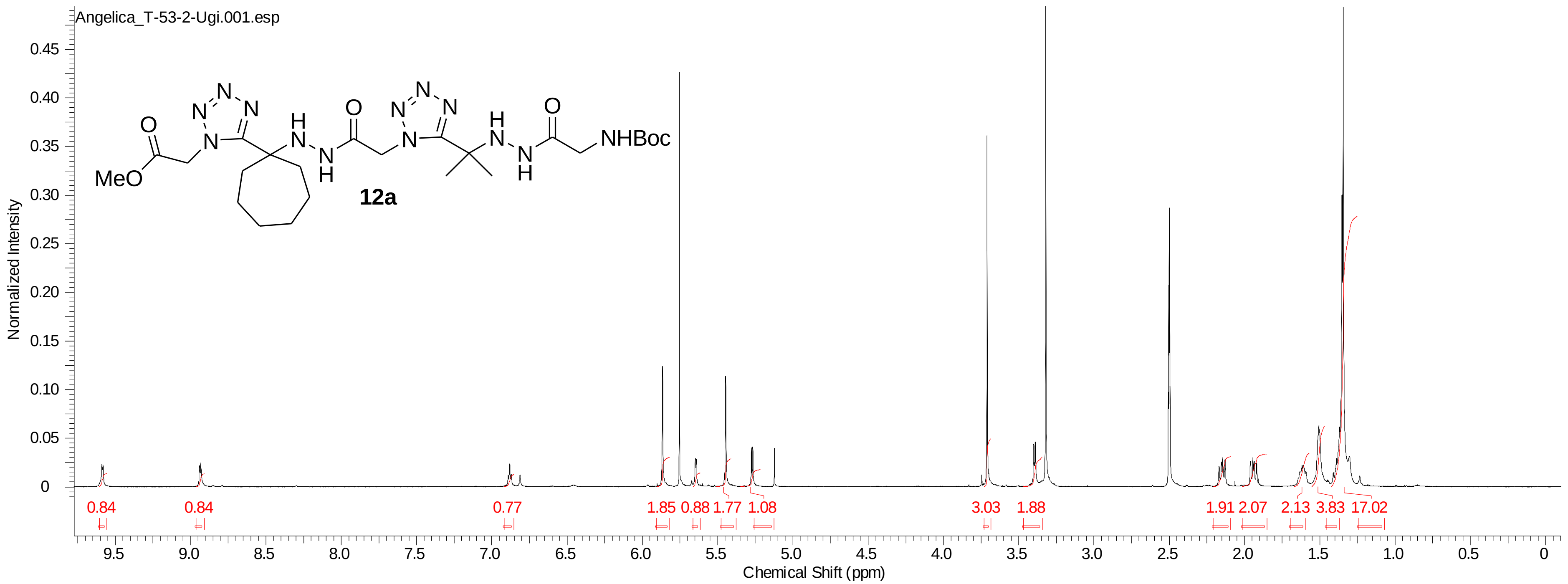

Figure S42: ^{1}H NMR (600 MHz, DMSO-d_6) spectrum of compound **12a**.

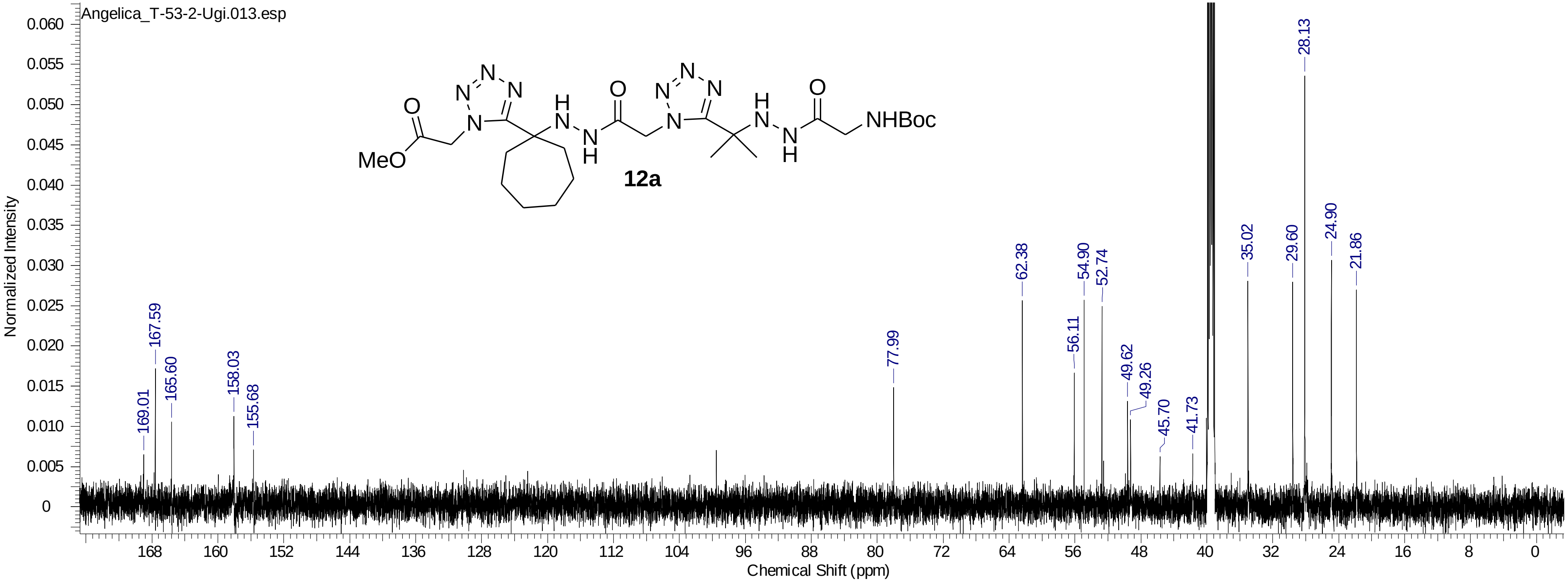

Figure S43: ^{13}C NMR (150 MHz, DMSO-d_6) spectrum of compound **12a**.

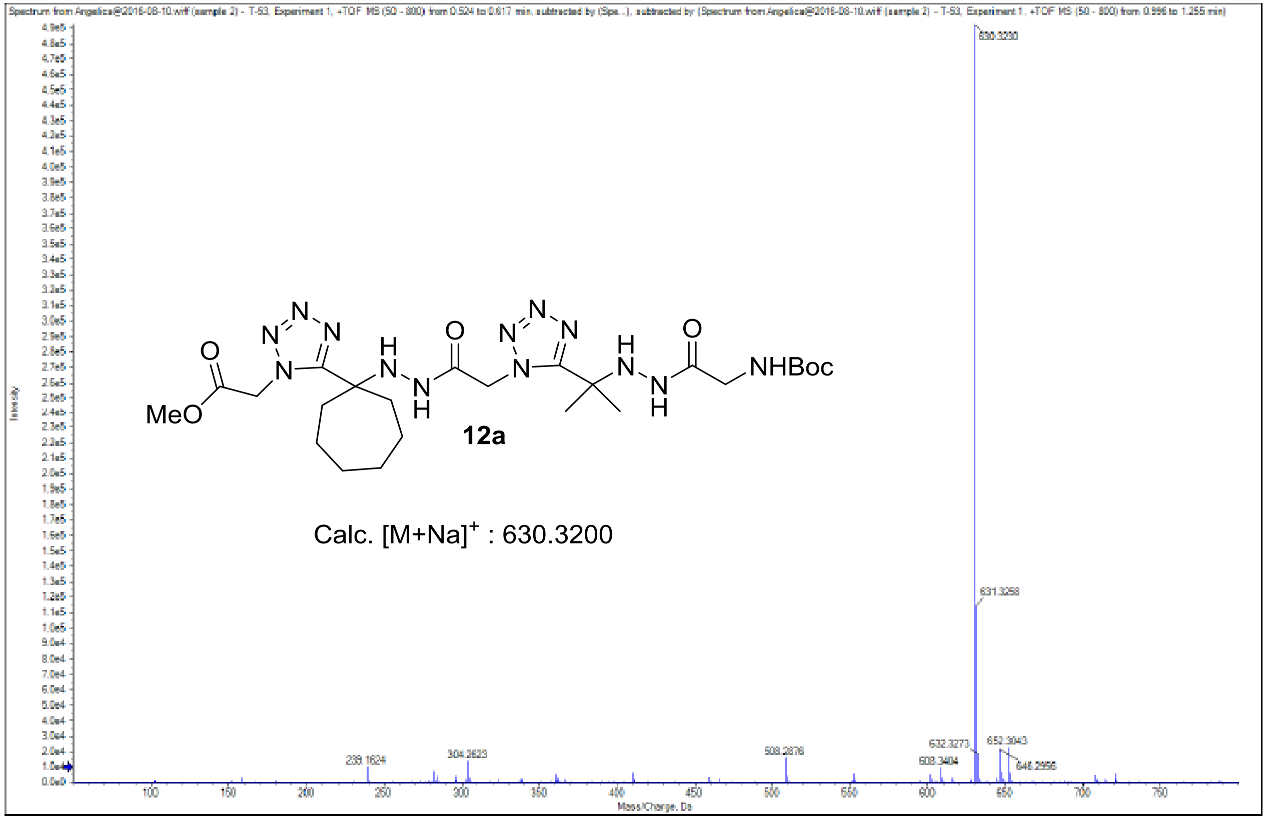

Figure S44: ESI-HRMS of compound **12a.**

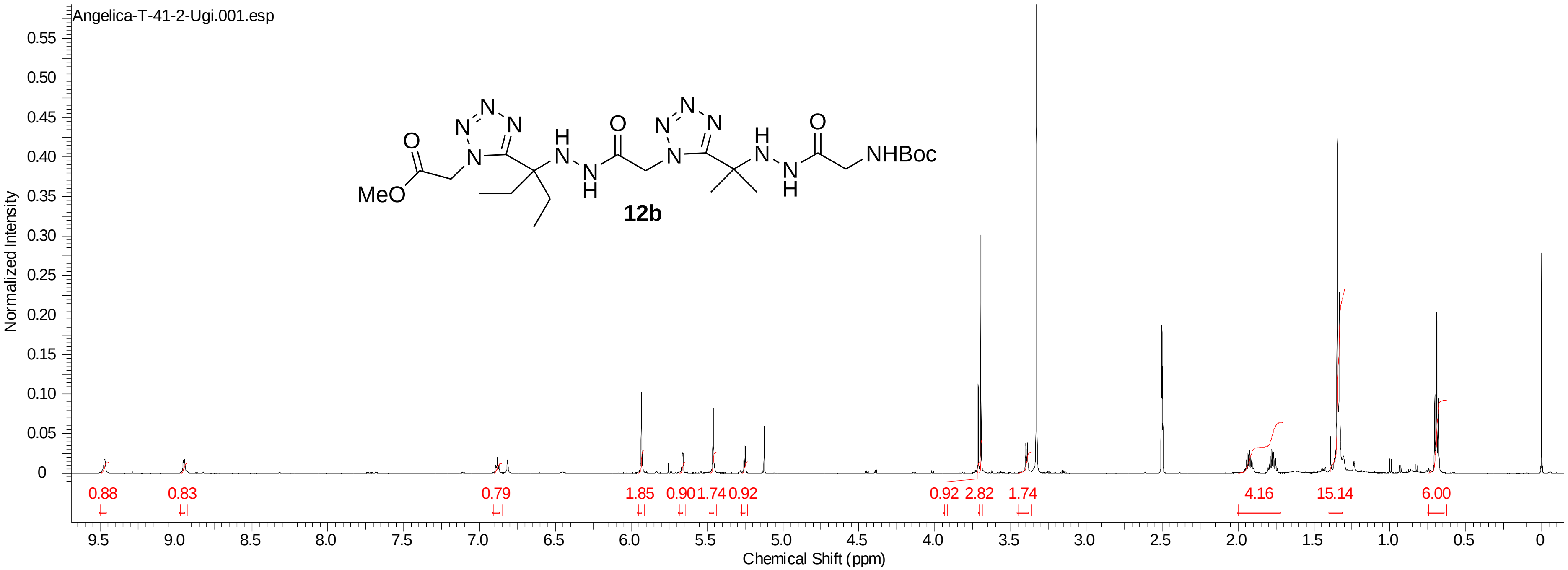

Figure S45: ^{1}H NMR (600 MHz, DMSO-d_6) spectrum of compound **12b**.

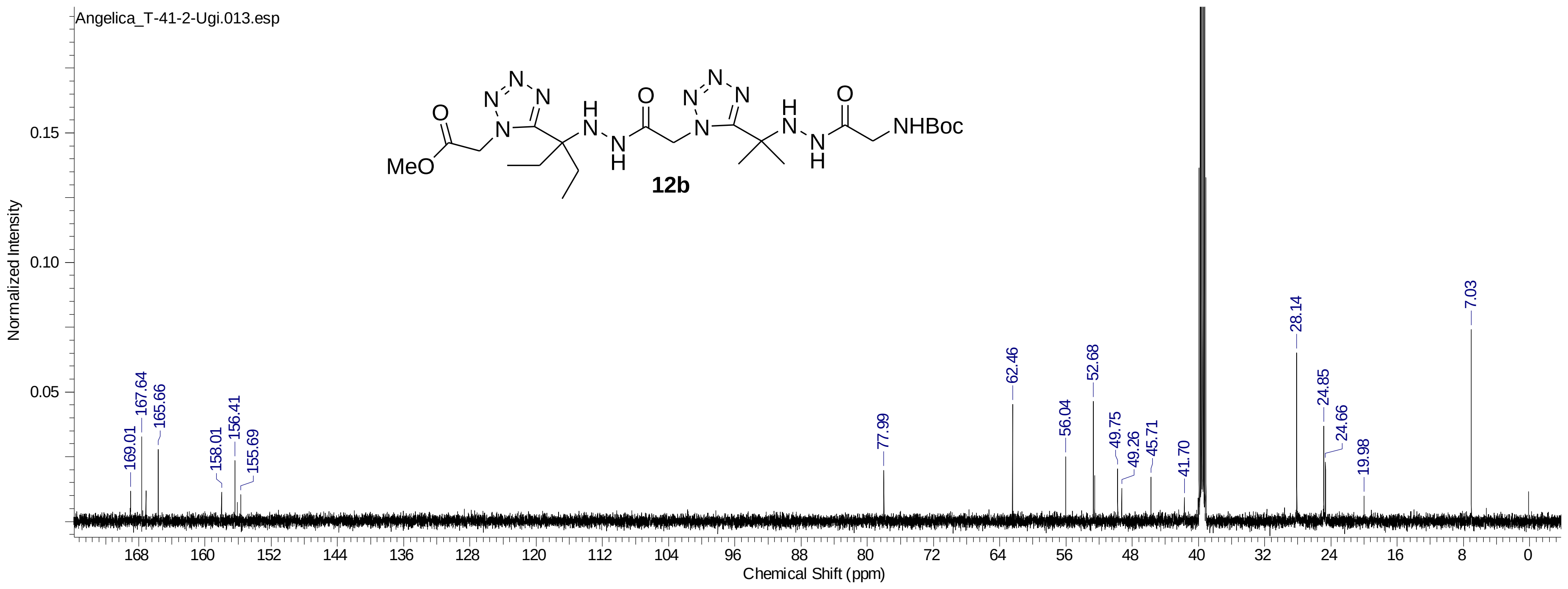

Figure S46: ^{13}C NMR (150 MHz, DMSO-d_6) spectrum of compound **12b**.

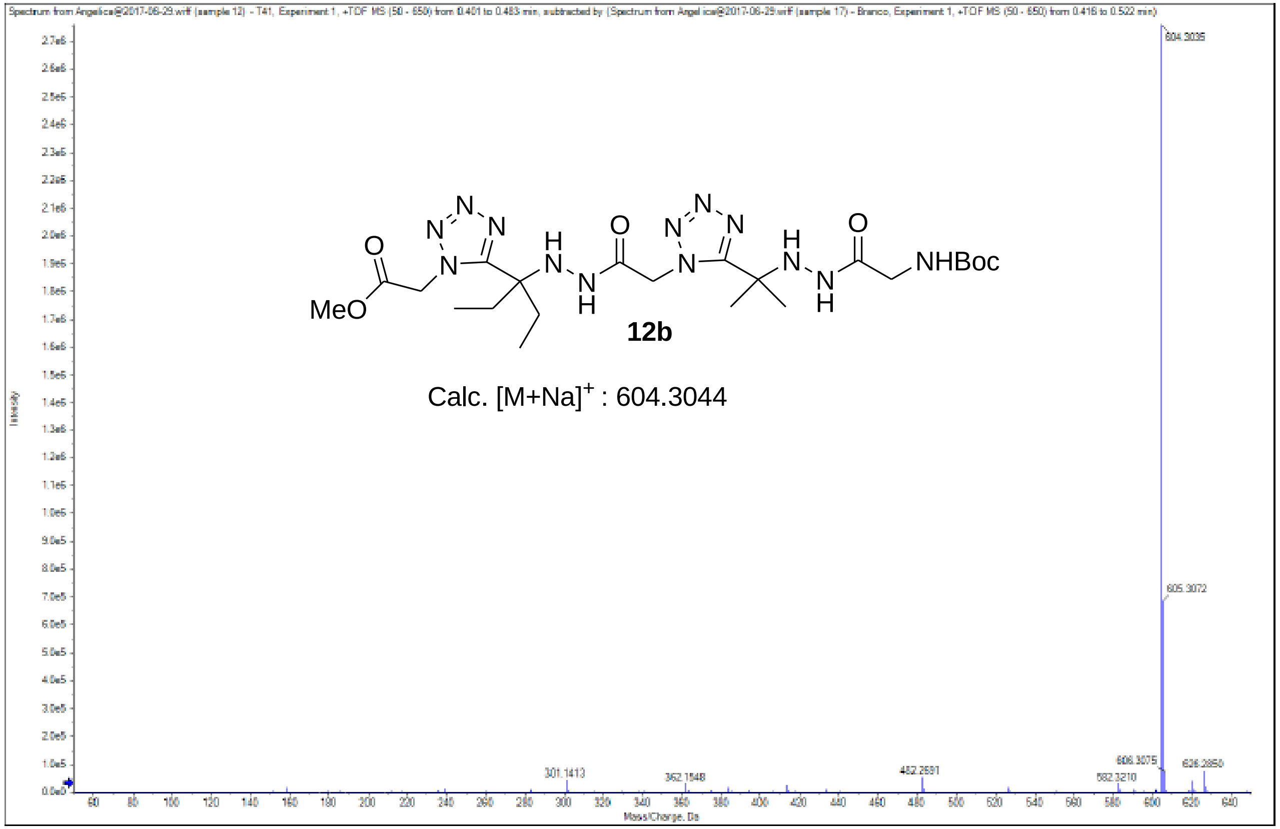

Figure S47: ESI-HRMS of compound **12b.**

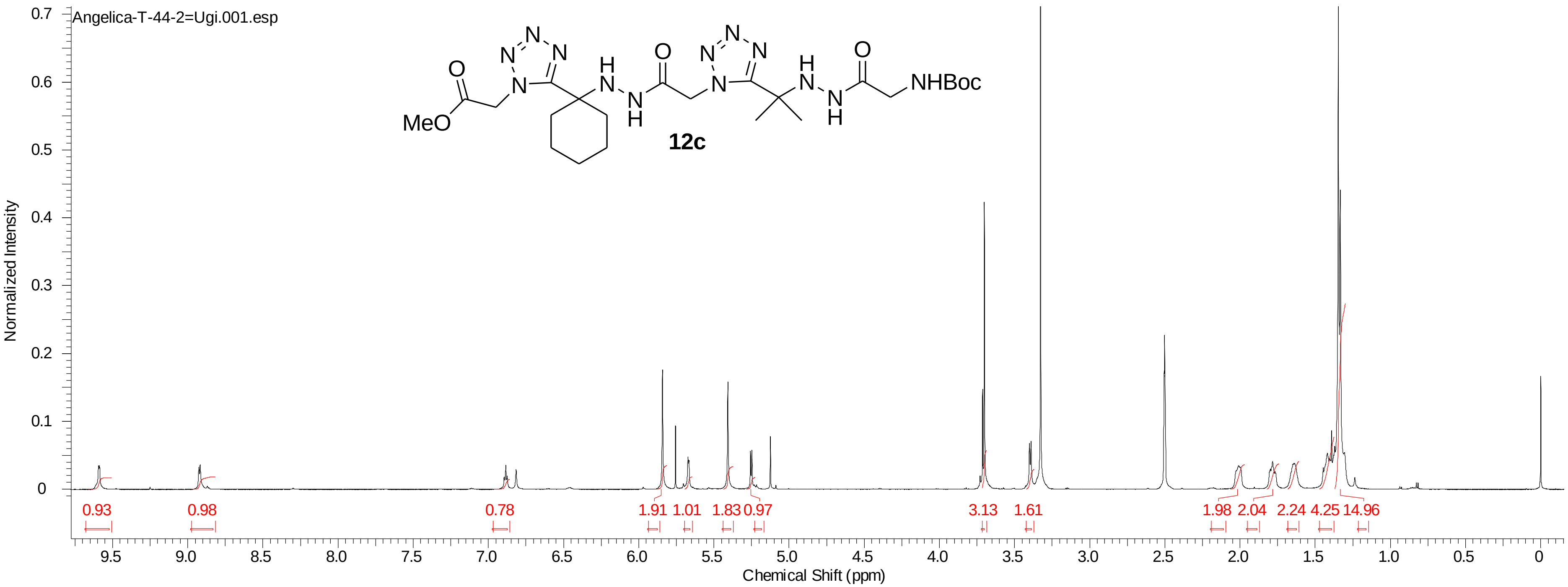

Figure S48: ^{1}H NMR (600 MHz, DMSO-d_6) spectrum of compound **12c**.

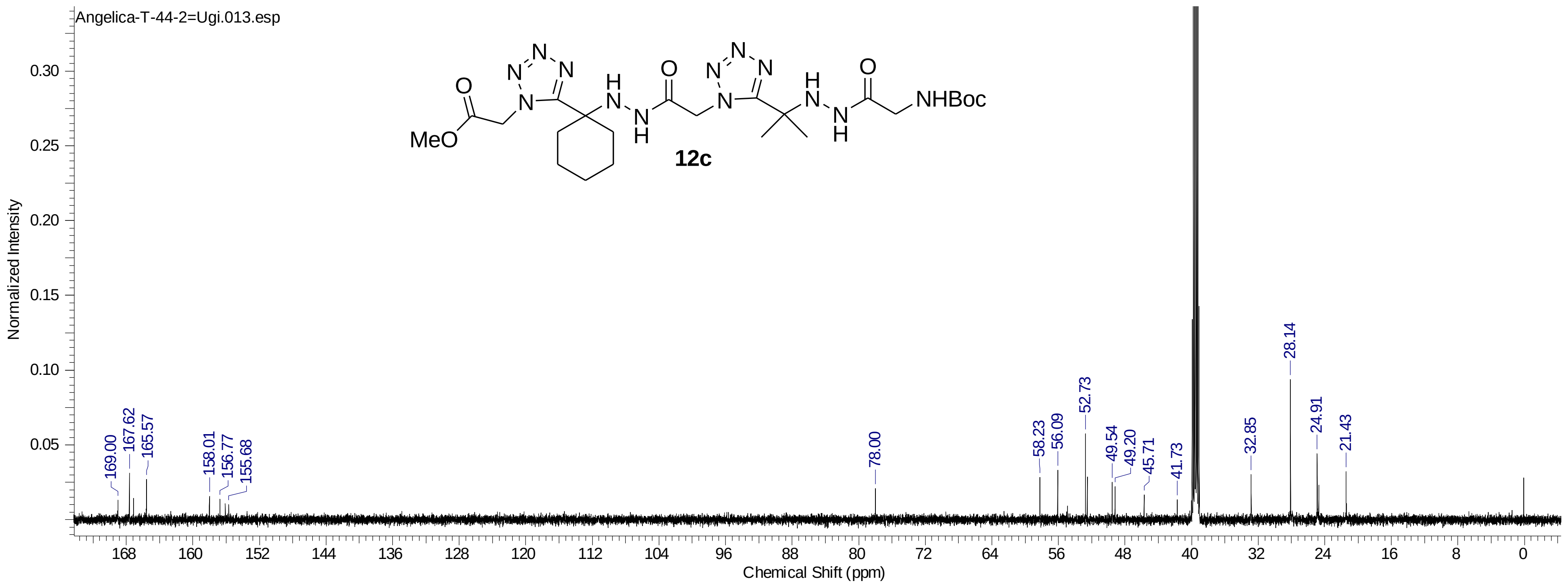

Figure S49: ^{13}C NMR (150 MHz, DMSO-d_6) spectrum of compound **12c**.

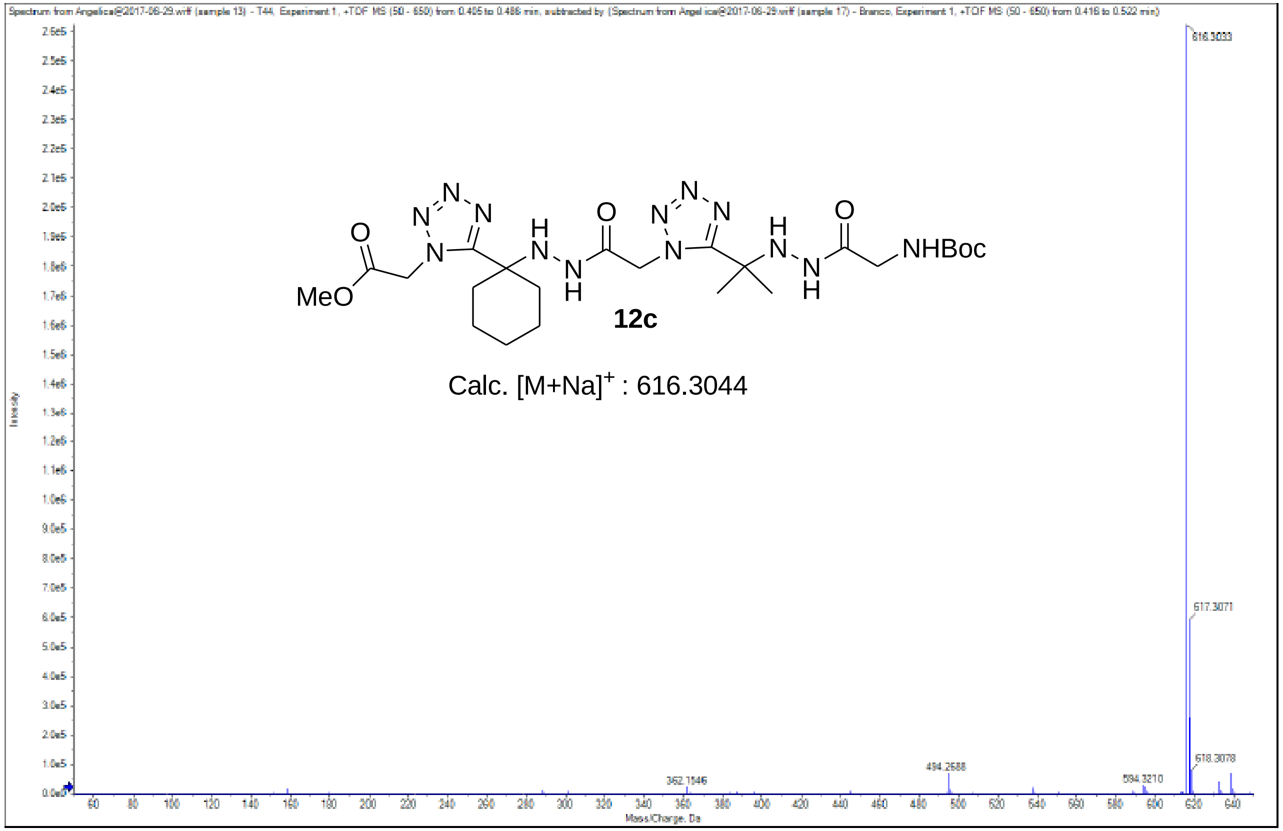

Figure S50: ESI-HRMS of compound **12c.**

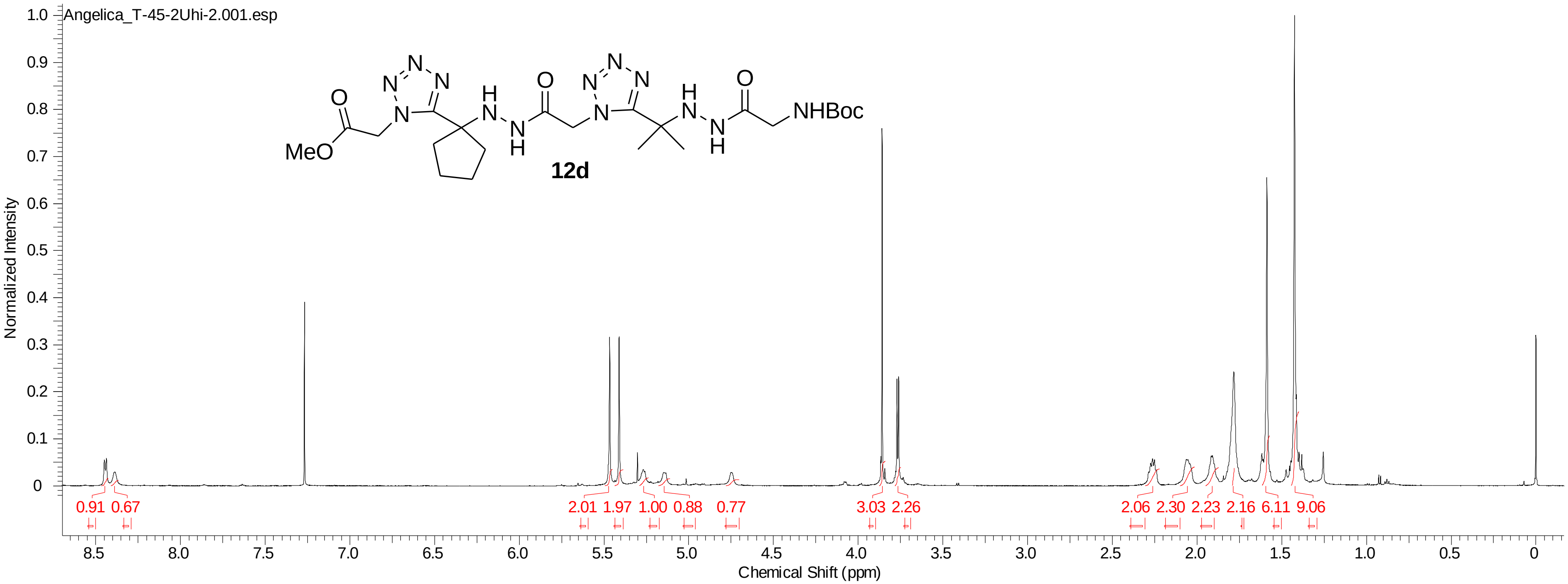

Figure S51: ^{1}H NMR (600 MHz, $CDCl_3$) spectrum of compound **12d**.

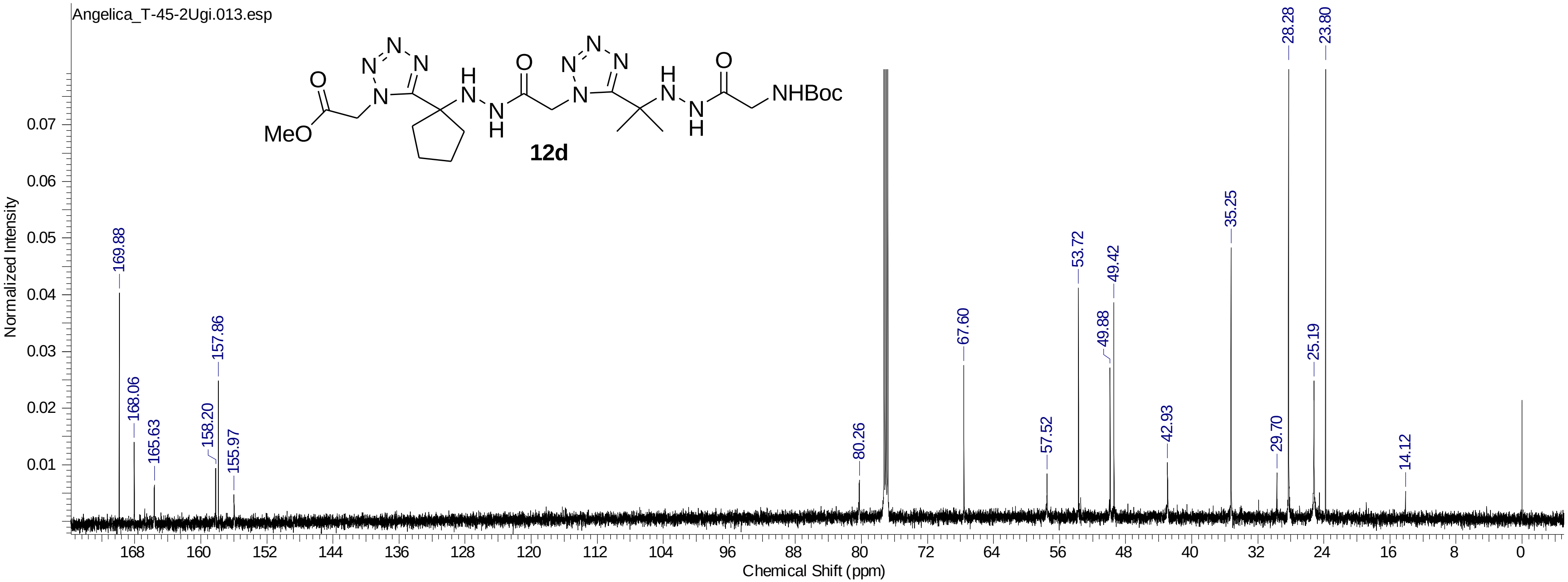

Figure S52: ^{13}C NMR (150 MHz, $CDCl_3$) spectrum of compound **12d**.

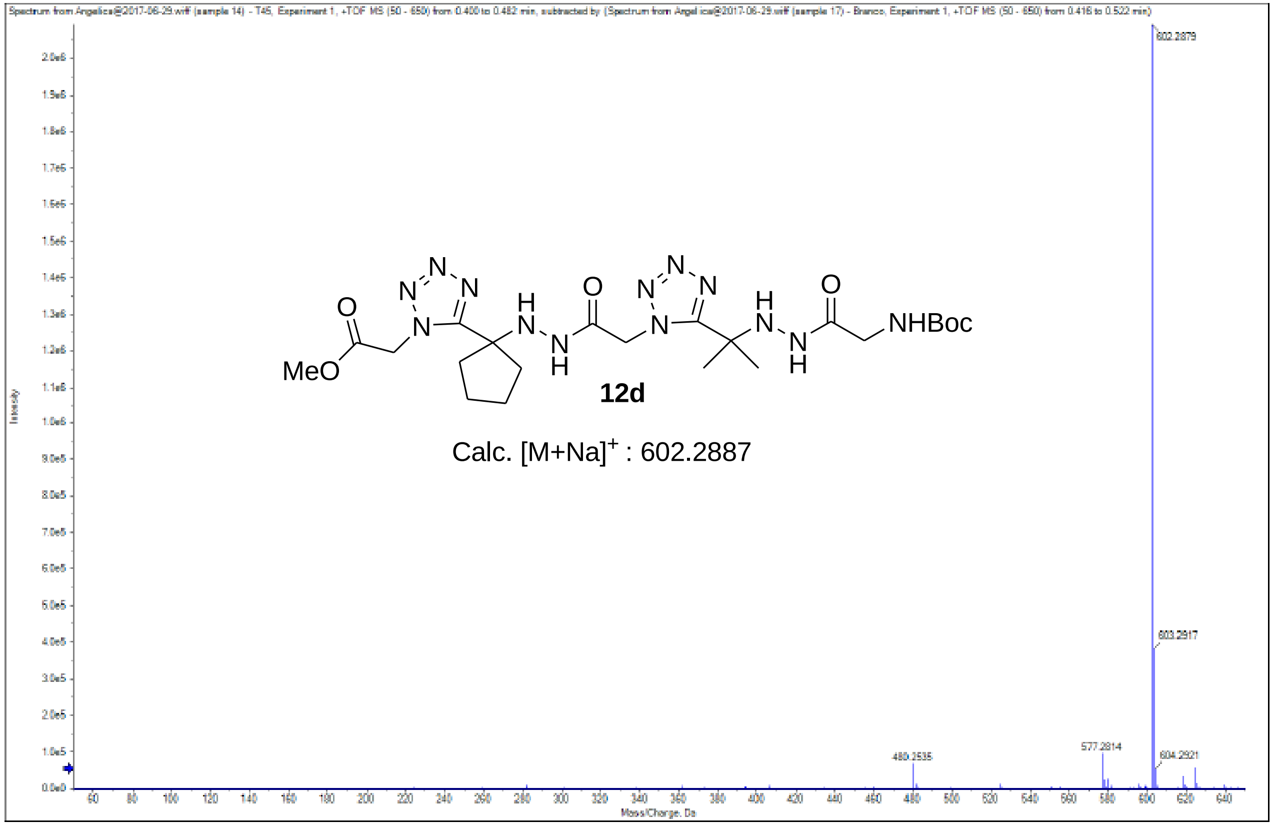

Figure S53: ESI-HRMS of compound **12d.**
